# CD4 T cell-intrinsic STING signaling controls the differentiation and effector functions of T_H_1 and T_H_9 cells

**DOI:** 10.1136/jitc-2021-003459

**Published:** 2022-01-28

**Authors:** Isis Benoit-Lizon, Elise Jacquin, Thaiz Rivera Vargas, Corentin Richard, Aurélie Roussey, Ludivine Dal Zuffo, Tiffany Martin, Andréa Melis, Daria Vinokurova, Sayyed Hamed Shahoei, Alvaro Baeza Garcia, Cassandre Pignol, Stéphane Giorgiutti, Raphaël Carapito, Romain Boidot, Frédérique Végran, Richard A Flavell, Bernhard Ryffel, Eric R Nelson, Pauline Soulas-Sprauel, Toby Lawrence, Lionel Apetoh

**Affiliations:** 1 INSERM, U1231, Dijon, France; 2 UFR Sciences de Santé, Université Bourgogne Franche-Comté, Dijon, France; 3 INSERM, UMR-S 1193, Université Paris-Saclay, Châtenay-Malabry, France; 4 Department of Molecular and Integrative Physiology, University of Illinois at Urbana Champaign, Urbana, IL, USA; 5 INSERM UMR - S1109, Department of Clinical Immunology and Internal Medicine, National Reference Center for Systemic Autoimmune Diseases (CNR RESO), Tertiary Center for Primary Immunodeficiency, Hôpitaux Universitaires de Strasbourg, Université de Strasbourg, Strasbourg, France; 6 Laboratoire d'ImmunoRhumatologie Moléculaire, GENOMAX platform, INSERM UMR_S 1109, Faculté de Médecine, Fédération Hospitalo-Universitaire OMICARE, Fédération de Médecine Translationnelle de Strasbourg (FMTS), LabEx TRANSPLANTEX, Strasbourg, France; 7 Department of Biology and Pathology of Tumors, Centre Georges François Leclerc, Dijon, France; 8 Department of Immunobiology, Yale University School of Medicine, New Heaven, CT, USA; 9 UMR 7355, Experimental and Molecular Immunology and Neurogenetics, CNRS, Orléans, France; 10 Department of Immunology, Institute of Infectious Disease and Molecular Medicine, University of Cape Town, Cape Town, South Africa; 11 Anticancer Discovery from Pets to People Theme, University of Illinois Urbana-Champaign, Cancer Center at Illinois, Urbana Champaign, Illinois, USA; 12 University of Illinois Cancer Center, University of Illinois at Chicago, Chicago, IL, USA; 13 INSERM UMR-S1109, Department of Clinical Immunology and Internal Medicine, National Reference Center for Systemic Autoimmune Diseases (CNR RESO), Tertiary Center for Primary Immunodeficiency, Faculty of Pharmacy, Université de Strasbourg, Strasbourg, France; 14 Centre d'Immunologie de Marseille-Luminy, Université Aix-Marseille, INSERM, CNRS, Marseille, France; 15 INSERM, U1100, Tours, France; 16 Faculté de Médecine, Université de Tours, Tours, France

**Keywords:** CD4-positive T lymphocytes, adaptive immunity, immunomodulation, melanoma

## Abstract

**Background:**

While stimulator of interferon genes (STING) activation in innate immune cells of the tumor microenvironment can result in CD8 T cell-dependent antitumor immunity, whether STING signaling affects CD4 T-cell responses remains elusive.

**Methods:**

Here, we tested whether STING activation modulated the effector functions of CD4 T cells in vivo by analyzing tumor-infiltrating CD4 T cells and evaluating the contribution of the CD4 T cell-derived cytokines in the antitumor activity of the STING ligand 2′3′-cyclic guanosine monophosphate-adenosine monophosphate (cGAMP) in two mouse tumor models. We performed ex vivo experiments to assess the impact of STING activation on CD4 T-cell differentiation and investigate the underlying molecular mechanisms. Finally, we tested whether STING activation enhances T_H_9 cell antitumor activity against mouse melanoma upon adoptive transfer.

**Results:**

We found that activation of STING signaling cell-intrinsically enhances the differentiation and antitumor functions of T_H_1 and T_H_9 cells by increasing their respective production of interferon gamma (IFN-γ) and interleukin-9. IRF3 and type I interferon receptors (IFNARs) are required for the STING-driven enhancement of T_H_1 cell differentiation. However, STING activation favors T_H_9 cell differentiation independently of the IFNARs/IRF3 pathway but through mammalian target of rapamycin (mTOR) signaling, underscoring that STING activation differentially affects the fate of distinct CD4 T-cell subsets. The therapeutic effect of STING activation relies on T_H_1 and T_H_9-derived cytokines, and STING activation enhances the antitumor activity of T_H_9 cells upon adoptive transfer.

**Conclusion:**

Our results reveal the STING signaling pathway as a therapeutic target to boost CD4 T-cell effector functions and antitumor immunity.

## Introduction

CD4 effector T cells can make decisive contributions to antitumor immunity. This is underscored not only by preclinical studies indicating that activation of CD4 T cells in the tumor microenvironment is required for productive antitumor immune responses and tumor clearance,^1^ but also by clinical investigations showing that the adoptive transfer of antigen-specific CD4 T cells into melanoma patients can lead to remission.^2^ Following the characterization of T_H_1 cells as interferon gamma (IFN-γ)-producing cells, which are differentiated from naive CD4 T cells in the presence of interleukin (IL)-12,^3^ the antitumor functions of these cells were exemplified in mouse and human cancers. More recently, the IL-9-secreting T_H_9 cells were characterized as another effector T-cell subset with potent antitumor properties. We and others have shown that these cells exert IL-9-mediated antitumor functions upon adoptive transfer in melanoma-bearing mice, identifying them as potential effector T cells for adoptive cell therapy of cancer.^4–6^ T_H_9 cells were originally shown to differentiate from naive CD4 T cells stimulated with transforming growth factor beta (TGF-β) and IL-4.^7^ Proinflammatory factors including IL-1β and tumor necrosis factor alpha (TNF-α) enhance T_H_9 differentiation.^5 8–10^ A growing number of studies also indicate that T_H_9 cells are essential for the efficacy of cancer immunotherapy treatments such as dendritic cell (DC) vaccination and anti-glucocorticoid-induced tumor necrosis factor receptor (GITR) therapy.^11 12^

The differentiation of naive CD4 T cells requires T-cell receptor (TCR)-driven, costimulatory, and cytokine-derived signals. These signals can be provided by activated innate immune myeloid cells such as DCs, which drive T-cell activation and polarization and thus dictate the magnitude and quality of adaptive immune responses.^13^ The detection of danger signals from invading viruses or bacteria or from damaged autologous cells drives DC maturation and cytokine secretion.^14^ The triggering of cytosolic receptors in innate immune cells leads to activation of the adaptor protein stimulator of interferon genes (STING),^15^ which results in the induction of a specific transcriptional program that culminates in the expression of type I interferons (IFNs) and proinflammatory cytokines.^16^ The administration of the STING ligand cyclic guanosine monophosphate-adenosine monophosphate (hereafter referred to as cGAMP) into solid tumors results in the induction of anticancer immune responses that promote tumor control.^17–19^ Likewise, the systemic delivery of a small-molecule STING agonist induces potent CD8 T cell-dependent anticancer immunity in vivo in mice.^20^ The clinical potential of STING targeting in solid tumors is currently being evaluated.^21^

While the functions of STING have been extensively studied in myeloid cells, we and others documented that STING is also expressed in CD4 T cells.^22–24^ Potent STING activation using synthetic ligands has been proposed to trigger T-cell death through type I-dependent and type I-independent pathways.^24–27^ However, the consequences of CD4 T cell-intrinsic STING activation on their differentiation and effector responses remain unclear. Here we report that the activation of STING in differentiating T_H_1 and T_H_9 cells enhances their respective secretion of IFN-γ and IL-9, resulting in enhanced anticancer activity in vivo. Our results uncover STING as a molecular target to modulate CD4 T-cell differentiation and functions for therapeutic use.

## Methods

### Mice

Wild type (WT) female C57BL/6 mice were purchased from Charles River laboratories (France). *Irf3*^−/−^ (IRF3^−/−^, RBRC00858 - RIKEN), *Rela*^fl/fl^ (p65, 024342, The Jackson Laboratory), Cd4-Cre (017336, The Jackson Laboratory), IL-9-Green Fluorescent Protein (GFP),^28^ Sting1^−/−^, OT-II (004194, The Jackson Laboratory), Trp1 (008684, The Jackson Laboratory), and *Ifnar*^−/−^ C57BL/6 mice were all bred at the Transgénèse et Archivage d’Animaux Modèles (TAAM, Orléans, France). IL-9-GFP as well as Sting1^−/−^ and type I interferon receptor (IFNAR)^−/−^ mice were respectively provided by Professor Richard Flavell and Professor Bernhard Ryffel. STING V154M/WT^29^ mice and control littermates were kindly provided by Professor Soulas-Sprauel.

For in vivo experiments, 6–12 week-old female mice were matched by age and randomly assigned to specific treatment groups except for Rag2^−/−^ STING^−/−^ experiments which include male mice equally distributed among the different treatment groups.

For in vitro experiments, 6–12 week-old mice were matched by age and sex. All transgenic mice used were on a C57BL/6 background and were age-matched with WT controls for experiments.

### Animal procedures

#### Cell lines and tumor growth experiments

B16-F10, B16-OVA (B16-F10 cells engineered to express OVA) mouse melanoma, MC38 and MC38-OVA (MC38 cells engineered to express OVA) mouse colon adenocarcinoma cell lines were cultured at 37°C under 5% CO_2_ in the following culture media. B16-F10 were maintained in Dulbecco's Modified Eagle Medium (DMEM) high glucose (Dutscher) supplemented with 10% (vol/vol) heat-inactivated fetal bovine serum (FBS, Dutscher) and 100 U/mL penicillin, 0.1 mg/mL streptomycin, 0.25 µg/mL amphotericin B (1%Penicillin Streptomycin Amphotericin B, PSA, Pan Biotech). B16-OVA, MC38 and MC38-OVA were respectively maintained in Roswell Park Memorial Institute (RPMI) 1640 w/L-glutamine or DMEM high glucose containing 10 mM Hepes (Gibco) supplemented with 10% (vol/vol) heat-inactivated FBS, 1% PSA, 1 mM sodium pyruvate (Gibco), 2 mM L-glutamine (Gibco) and Minimum Essential Medium- Non-Essential Amino Acids (MEM NEAA) (0.1 mM each AA, Gibco). B16-F10 and MC38 cells were respectively obtained from American Type Culture Collection (ATCC) and Kerafast. B16-OVA and MC38-OVA cells were respectively kindly provided by Professor Yong Lu (Department of Microbiology and Immunology, Wake Forest School of Medicine, Winston-Salem, North Carolina, USA) and Professor Ana Anderson (Harvard Medical School, Boston, USA).

Tumor cells (1.5×10^5^ B16-F10 or B16-OVA cells per mouse, 5×10^5^ MC38 or MC38-OVA cells per mouse) were resuspended in sterile phosphate buffered saline (PBS) and implanted subcutaneously. Each mouse received two intratumoral injections of PBS containing 100 µg of cGAMP (G(2’,5’)pA(3’,5’)p, Invivogen) encapsulated in 2,5 µL of Lipofectamine 2000 (Invitrogen) or PBS with Lipofectamine 2000 alone (control) on days 5 and 10.

As for the Rag2^−/−^ STING^−/−^ experiment, mice were injected intravenously with 5×10^6^ WT or STING^−/−^ CD4 T cells and 2.5×10^6^ STING^−/−^ CD8 T cells, respectively, isolated with CD4 (L3T4) and CD8 (Ly-2) MicroBeads (Miltenyi Biotec) according to the manufacturer’s instructions. MC38 tumor implantations were performed 5 weeks post-T cell transfer and mice were treated as described above.

Tumor length and width were measured three times a week using calipers. Mice with tumor size exceeding 300 mm^2^ or with ulcerated tumors were euthanized in agreement with ethical guidelines.

Alternatively, for tumor-infiltrating lymphocyte (TIL) analysis, MC38 tumor-bearing mice were treated intratumorally when tumors reached 40–50 mm² with cGAMP (50 µg per mouse) or PBS with Lipofectamine 2000 alone (control) and tumors were harvested 1 day after treatment.

As for TIL analysis in MC38-OVA tumor-bearing mice, mice were injected intravenously with OT-II CD4 T cells (6×10^6^) 4 days before MC38-OVA subcutaneous injection.

#### Cytokine neutralization

Cytokine neutralization was achieved by intraperitoneal injections (200 µg per mouse) of anti-IFN-γ (clone XMG1.2, BioXCell), anti-IL-9 (clone 9C1, BioXCell), anti-IL-4 (clone 11B11, BioXCell) or anti-IL-17 (clone 17F3, BioXCell) antibodies 1 day before tumor cell implantation and then three times a week. Alternatively, IFN-γ neutralization in MC38 tumor-bearing mice was achieved by intraperitoneal injections (200 µg per mouse) of anti-IFN-γ on days 4, 5, 7, 9, 10, 12, and 14 after tumor cell implantation. As control for anti-IL-17 or anti-IFN-γ, anti-IL-4, and anti-IL-9, rat IgG1 isotype control (clone HRPN, BioXCell), mouse IgG1 isotype control (clone MOPC-21, BioXCell), and mouse IgG2a isotype control (clone C1.18.4, BioXCell) were respectively used.

#### Adoptive transfer of T_H_9 cells

B16-OVA tumor-bearing mice (5 days after subcutaneous tumor implantation) were injected intravenously with 2×10^6^ effector OT-II T_H_9 cells stimulated or not with cGAMP before in vitro polarization. Tumor length and width were measured three times a week using calipers. Mice with tumor size exceeding 300 mm^2^ or with ulcerated tumors were euthanized in agreement with ethical guidelines.

Alternatively, 2.5×10^5^ B16-OVA cells per mouse were injected intravenously in the tail vein. One day after, mice received intravenous injections of 2×10^6^ effector OT-II T_H_9 stimulated or not with cGAMP before in vitro polarization. Mice were euthanized 14 days after T_H_9 cell adoptive transfer and lung tumor foci were enumerated in a blinded manner.

B16-F10 tumor bearing mice (7 days after subcutaneous tumor implantation) were first injected intraperitoneally or not with cyclophosphamide (CTX, 200 mg/kg) and the next day injected intravenously with 1×10^6^ effector Trp1 T_H_9 cells. Trp1 T_H_9 cells were obtained by coculture of naive Trp1 CD4 T cells with 5 µg/mL of Trp1_106-133_ major histocompatibility complex (MHC) class II-restricted TRP1 (SGHNCGTCRPGWRGAACNQKILTVR) (GenScript)) peptide-loaded STING^−/−^ antigen-presenting cell (APC) (splenocytes depleted from CD4 and CD8 T cells using magnetic enrichment, 1 APC:10 CD4 T-cell ratio) and stimulated or not with cGAMP before in vitro polarization as described further. Tumor length and width were measured three times a week using calipers. Mice with tumor size exceeding 300 mm^2^ or with ulcerated tumors were euthanized in agreement with ethical guidelines.

### Ex vivo procedures

#### TIL analysis

MC38 tumors were subjected to mechanic dissociation and enzymatic digestion using the Tumor Dissociation Kit mouse and the gentleMACS Dissociator (Miltenyi Biotec) according to the manufacturer’s instructions. The single-cell suspension was then washed two times using RPMI-1640 supplemented with 10% FBS, 1% PSA and 10 mM Hepes (hereafter referred to as complete RPMI). Immune cells were then enriched using CD45 TILs kit (Miltenyi Biotec). Half of the cells were stained with Fixable Viability Dye eFluor 780 (eBioscience), CD45-BV421 (30 F-11, BD Bioscience), and CD4-APC (clone RM4-5, BD Bioscience) for live CD4 T cells sorting using the BD FACSAria-III (BD Biosciences). Sorted CD4 T cells were directly lysed in TriReagent (Ambion) or lysis buffer (Quick-RNA MicroPrep Kit Zymo Research) for RNA analysis.

Remaining cells were then stimulated using Cell Stimulation Cocktail (eBioscience) and Protein Transport Inhibitor Containing Monensin (PTI, eBioscience) diluted in complete RPMI supplemented with 1 mM sodium pyruvate, 2 mM L-glutamine, MEM NEAA (0.1 mM each AA) and 50 µM 2-mercaptoethanol (Gibco)—hereafter referred to as mouse T-cell culture medium—for 2 hours at 37°C under 5% CO_2_ and subjected to immunostaining and flow cytometry analysis.

As for TIL analysis in MC38-OVA tumor bearing mice, tumor-infiltrating immune cells were restimulated with class II-restricted OVA-peptide (323-339) for 3 hours with Protein Transport Inhibitor Containing Monensin (PTI, eBioscience).

Cell viability was assessed using the LIVE/DEAD Fixable Blue Dead Cell Stain Kit, for UV excitation (Invitrogen) or Fixable Viability Dye eFluor 780 (eBioscience). Extracellular markers were stained with the following antibodies: CD45 APC-Vio770 (clone REA737, Miltenyi Biotec) or CD45-BV421 (clone 30 F-11, BD Bioscience), CD4 PercP-Vio700 (clone REA604, Miltenyi Biotec), and CD8 PE-Vio770 (clone REA601, Miltenyi Biotec). After fixation and permeabilization using the Fixation/Permeabilization Solution Kit (BD Biosciences) according to the manufacturer’s instructions, intracellular staining of IFN-γ and IL-9 was performed using an anti-mouse IFN-γ APC or PE antibody (clone AN.18.17.24 or REA638, Miltenyi Biotec) and mouse IL-9 APC or BV421 antibody (clone RM9A4, BD Bioscience), respectively. Single-cell suspensions were analyzed by flow cytometry using BD LSR FORTESSA cytometer (BD Biosciences) equipped with BD FACSDiva software (BD Biosciences). Flow cytometry data were then analyzed using Flowlogic software (Miltenyi Biotec). Gating strategies are presented in online supplemental figure S1A.

10.1136/jitc-2021-003459.supp1 Supplementary data

#### Mouse primary cell isolation

Naive CD4 T cells (CD4^+^CD62L^hi^) were isolated from mouse spleen and lymph nodes. CD4 T cells were first enriched with anti-CD4 microbeads (L3T4) (Miltenyi Biotec) according to the manufacturer’s instructions and then further stained with CD4 Vioblue (clone REA604, Miltenyi Biotec), CD62L PE (clone MEL-14, BD Biosciences) antibodies before sorting using the BD FACSAria-III (BD Biosciences). Isolated naive T cells were routinely 98% pure. Alternatively, for western blots (WBs) and adoptive transfer experiments, naive CD4 T cells were purified using a naive CD4 T-cell isolation kit (Miltenyi Biotec).

Total effector and memory CD4 T cells from STING V154M/WT mice and their WT littermates were isolated with a mouse CD4^+^ T-cell isolation kit (Miltenyi Biotec) and directly restimulated using Cell Stimulation Cocktail and PTI diluted in mouse T cell culture medium for 2 hours at 37°C under 5% CO_2_ and subjected to immunostaining using LIVE/DEAD Fixable Blue Dead Cell Stain Kit, for UV excitation, CD4 FITC (clone RM4-4, BD Biosciences) or CD4 PercP-Vio700 (clone REA604, Miltenyi Biotec) and CD45 APC-Vio770 (clone REA737, Miltenyi Biotec) antibodies. IFN-γ intracellular staining was performed using the Fixation/Permeabilization Solution Kit (BD Biosciences) and IFN-γ PE antibody (clone REA638, Miltenyi Biotec). Cells were analyzed using a BD LSRFORTESSA cytometer equipped with BD FACSDiva software and data were analyzed using FlowLogic software. Gating strategies are presented in online supplemental figure S1B.

Total CD8 cells were isolated from STING^−/−^ mouse spleen and lymph nodes using anti-CD8 microbeads (Ly-2) (Miltenyi Biotec) according to the manufacturer’s instructions.

#### Human T-cell isolation

Human naive CD4 T cells (CD4^+^ CD45RA^+^) were isolated from healthy donor blood. CD4 T cells were purified using RosetteSep Human CD4^+^ T Cell Enrichment Cocktail (StemCell) according to the manufacturer’s instructions and then further stained with CD4 BV605 (clone RPA-T4, BioLegend) and CD45RA PE/APC (clone HI100, BioLegend) antibodies before sorting using the BD FACSAria-III.

#### Cell transfection and treatments

Mouse naive CD4 T cells were seeded on plate-bound anti-CD3 (2 µg/mL, clone 17A2; BioXCell) and anti-CD28 (2 µg/mL, clone PV1; BioXCell). Human naive CD4 T cells were seeded on plate-bound anti-CD3 (5 μg/mL, clone OKT-3; BioXCell) and anti-CD28 (2 µg/mL, clone CD28.2; BioLegend). Cells (3×10^6^ cells/mL) were transfected for 6 hours with 80 µg/L cyclic dinucleotides (unless specified otherwise) using Opti-MEM Glutamax (Gibco) and Lipofectamine 2000 (Invitrogen), according to the manufacturer’s instructions. Alternatively, 1.5×10^6^ cells/mL CD4 T cells were treated with indicated doses of 5,6-dimethylxanthenone-4-acetic acid (DMXAA) for 4 hours in mouse T-cell culture medium.

2′3′-cGAMP (cyclic (G(2′,5′)pA(3′,5′)p)), referred to as cGAMP; 2′3′-cGAMP control (2′5′-GpAp), referred to as control; as well as DMXAA (Murine STING ligand, Xanthenone Analog) were purchased from InvivoGen.

When indicated, mTOR inhibition was achieved by adding 10 nM of rapamycin (Rapa; Calbiochem, Merck) during the 6-hour transfection step.

#### T-cell in vitro polarization

After cell transfection and/or treatment, supernatants were replaced by mouse T-cell culture medium or AIM V medium (Gibco) for human cells containing polarization cytokines as follows: anti-mouse IFN-γ (50 μg/mL, clone XMG 1.2; BioXCell) and anti-mouse IL-4 (50 μg/mL, clone 11B11; BioXCell) for mouse T_H_0 cells; IL-12 (10 ng/mL, Miltenyi Biotec) and anti-mouse IL-4 for mouse T_H_1 cells; TGF-β (2 ng/mL, Miltenyi Biotec), IL-4 (20 ng/mL, Miltenyi Biotec), and anti-mouse IFN-γ for mouse T_H_9 cells; IL-6 (20 ng/mL, Miltenyi Biotec), TGF-β, anti-mouse IFN-γ, and anti-mouse IL-4 for mouse T_H_17, IL-12 (10 ng/mL, R&D System) and anti-human IL-4 (3.5 μg/mL, clone MP4-25D2; BioXCell) for human T_H_1 cells, TGF-β (5 ng/mL, Miltenyi Biotec), IL-4 (10 ng/mL, R&D System), and anti-human IFN-γ (3.5 μg/mL, clone NIB42; BioLegend) for human T_H_9 cells. Unless specified otherwise, cells were cultured for 3 days at 37°C under 5% CO_2_.

### IFN-γ, IL-17, and IL-9 intracellular level measurement

T_H_1 and T_H_17 cells were restimulated using Cell Stimulation Cocktail and PTI diluted in mouse T-cell culture medium for 2 hours at 37°C under 5% CO_2_. All cells were stained with Fixable Viability Dye eFluor 780 (eBioscience) or LIVE/DEAD Fixable Blue Dead Cell Stain Kit, for UV excitation and CD4 Vioblue (clone REA604, Miltenyi Biotec). IL-9-GFP expression in T_H_9 cells was assessed by flow cytometry directly after extracellular staining. As for T_H_1 and T_H_17 cells, IFN-γ and IL-17 intracellular staining was performed using the Fixation/Permeabilization Solution Kit (BD Biosciences) and anti-IFN-γ APC antibody (clone AN.18.17.24, Miltenyi Biotec) or anti-IL-17 BV605 (clone TC11-18H10, BD Bioscience), respectively. Cells were analyzed using a BD FACSCANTO cytometer or a BD LSRFORTESSA cytometer equipped with BD FACSDiva software, and data were analyzed using FlowLogic software. Gating strategies are presented in online supplemental figure S1C–E.

### Real-time quantitative PCR

Total RNA was extracted from T cells with TriReagent (Ambion) or Quick-RNA MicroPrep Kit (Zymo Research). Depending on experiments, 10–500 ng of total RNA was retrotranscribed using M-MLV Reverse Transcriptase (Invitrogen) or with iScript cDNA Synthesis Kit (Bio-Rad). cDNA was analyzed by real-time quantitative PCR with Power SYBR Green PCR Master Mix or PowerUp SYBR Green Master Mix (Applied Biosystems) or iTaq Universal SYBR Green Supermix 5000 (Bio-Rad) according to the manufacturer’s instructions using the ViiA 7 Real-Time PCR System (Applied Biosystems) combined with 500 nM of forward and reverse primers (primer sequences are indicated in online supplemental table S1).

Gene expression in CD4 T cells isolated from STING V154M/WT mice and MC38 TILs was analyzed using the TaqMan Fast Advanced Master Mix (Applied Biosystems) and TaqMan Gene Expression Assay (Applied Biosystems) according to the manufacturer’s instructions. A list of TaqMan assays is depicted in online supplemental table S1. As for gene expression in CD4 T cells isolated from MC38 TILs, equivalent of 6 ng of cDNA were preamplified with TaqMan PreAmp Master Mix (Applied Biosystems) combined with pooled TaqMan Gene Expression Assays in a final volume of 25 µL and according to the manufacturer’s instructions.

Expression of target genes was normalized to the expression of mouse *Actb* or human *ACTB* (relative expression (RE)). When indicated, the fold change (FC) in RE was calculated by normalizing data to control conditions (WT cells treated with control, for each time point). When Rapa was used, FCs were calculated by normalizing data to each control condition of Rapa or vehicle treatment.

### Cytokine measurement

Cell culture supernatants were analyzed by ELISA for mouse IFN-γ (BD Bioscience), IL-9 (BioLegend), IL-17 (BioLegend) or IFN-β (PBL Assay Science) or for human IFN-γ (BioLegend) or human IL-9 (BioLegend) according to the manufacturer’s instructions.

### Western blot

Total protein extracts were obtained by lysing CD4 T cells into ice-cold RIPA Buffer (Pierce) containing Protease Inhibitor Cocktail (Sigma) and Phosphatase Inhibitor Cocktail 3 (Sigma) on ice for 10 min. Lysates were centrifuged at 13,000 *g* at 4°C for 10 min, and supernatants were retrieved for WB analyses. For subcellular fractionation, cytosolic (Cyt) and nuclear (Nuc) fractions were purified using the ProteoExtract Subcellular Proteome Extraction Kit (Merk) according to manufacturer’s instructions. Protein lysates were separated on 4%–15% gradient precast gels (Bio-Rad) before transfer on polyvinylidene difluoride membranes using a MIXED MW program on the Trans-Blot Turbo Transfer System (Bio-rad). Membranes were blocked in tris-buffered saline (TBS) containing 0.1% Tween 20 (TBS-T) and supplemented with 5% bovine serum albumin (BSA) or non-fat dry milk for 1 hour at room temperature and incubated overnight at 4°C with primary antibodies diluted in the appropriate blocking buffer. They were then incubated with a horseradish peroxidase-conjugated secondary antibody (Cell Signaling Technology (CST), 7074S) diluted 1:5000 in TBS-T 5% BSA and proteins were detected using enhanced chemiluminescence (Clarity Western ECL Substrate, Bio-Rad or SuperSignal West Femto Maximum Sensitivity Substrate, ThermoFisher). Densitometry analysis was performed using ImageJ software.

The following antibodies were purchased from Cell Signaling Technology (CST) and diluted 1:1000 in TBS-T 5% BSA: Phospho-STING (Ser 366, 19781), TBK1/NAK (clone D1B4, 3504) Phopho-TBK1 (Ser 172, clone D52C2, 5483), Phospho-IRF-3 (Ser 396, clone 4D4G, 4947), IRF3 (clone D83B9, 4302), Phospho-NF kappa B P65 (Ser 536, clone 93H1, 3033), NF kappa B P65 (clone D14E12, 8242), Phospho-p70S6K (Thr389, clone 108D2, 9234), and Phospho-S6 (Ser235/236, clone D57.2.2E, 4858). Antibody against histone H3 (clone D1H2, 4499, CST) was diluted 1:2000 in TBS-T 5% milk. Antibodies against β-actin horseradish peroxidase (HRP) conjugate (clone 8H10D10, 12262, CST) and anti-GAPDH HRP conjugate (clone 14C10, 3683, CST) were diluted 1:5000 in TBS-T 5% BSA. Antibody against TMEM173 (19 851–1-AP, Proteintech) was diluted 1:1000 in TBS-T 5% milk.

### RNAseq

#### STING V154M/WT mouse CD4 T-cell RNAseq analyses

Mouse splenocytes from STING V154M/WT mice (n=5) and control littermates (n=5) were stained with anti-CD3-FITC (clone 145–2 C11, BD Biosciences), anti-CD4-AF700 (clone RM4-5, BD Biosciences), and DAPI (Sigma) before positive sorting of live CD3^+^ CD4^+^ T cells using a BD FACSAria Fusion cell sorter (IGBMC Flow Cytometry Facility, Strasbourg, France). Isolated cells were at least 95% pure. Total RNA was extracted using the RNeasy Plus Micro Kit (Qiagen) according to the manufacturer’s instructions. RNAseq analyses were performed by the Genomax Facility (INSERM U1109, ImmunoRhumatologie Moléculaire, Université de Strasbourg, France). Libraries were prepared from 10 ng RNA using SMARTer Stranded Total RNA-Seq Kit, Pico Input Mammalian (Takara) following the manufacturers’ instructions. Briefly, random primers were used for first-strand synthesis, and ribosomal cDNA was cleaved by ZapR V.2 in the presence of mammalian R-probes V.2. Libraries were pooled and sequenced (paired-end 2×75 bp) on a NextSeq500 using the NextSeq 500/550 High Output Kit V.2 according to the manufacturer’s instructions (Illumina). For each sample, quality control was carried out and assessed with the next-generation sequencing (NGS) Core Tools FastQC. Reads were aligned against Mus musculus mm10 reference genome using TopHat 2 Aligner,^30^ and gene expression levels were estimated using Cufflinks V.2.1.1.^31^ Differential expression analysis was performed with Cuffdiff V.2.1.1 after exclusion of fragments per kilobase million below 9.

#### cGAMP-stimulated mouse T_H_1 and T_H_9 cell RNAseq analyses

Mouse naive CD4 T cells were isolated and treated with cGAMP or control and polarized in vitro into T_H_1 or T_H_9 cells as described previously for 16 and 48 hours. Total RNA was extracted with TriReagent according to the manufacturer’s instructions. Libraries were prepared from 500 ng of total RNA using TruSeq Stranded Total RNA Library Prep kit (Illumina) after rRNA removal with Ribo-zero rRNA Removal Kit (Illumina) following the manufacturers’ instructions. RNA sequencing was performed on NextSeq500 device (Illumina). The RNA-seq libraries were sequenced with single-end 76 bp reads. Raw FASTQ files were trimmed for residual adapter sequences and quality filtered through Trimmomatic software.^32^ Reads were pseudo-aligned against mm10 genome through Kallisto software and differential expression analysis was performed with DESeq2 R package.^33^ Unsupervised hierarchical clustering of genes was performed by using Clustvis web tool using Ward correlation. Gene set enrichment analysis (GSEA) was performed using GSEA Software^34 35^ and using EnrichR web tool^36 37^ with GO Biological Process 2018 database. Data are deposited in the Gene Expression Omnibus database under accession number GSE147300.

### Statistical analysis

Statistical analyses were performed using Prism software (Graph Pad software, La Jolla, California, USA). For two-group comparisons, Student t-tests was used. For multiple group comparison, ordinary one-way analysis of variance (ANOVA) with Dunnett’s multiple comparisons test or ordinary two-way ANOVA with Sidak’s multiple comparisons test was used. For survival curve statistics, log-rank (Mantel-Cox) test was used. All p values are two tailed.

## Results

### STING activation enhances CD4 T-cell effector functions in vivo

We investigated the immune-related mechanisms that contribute to the anticancer efficacy of cGAMP in vivo. For this, we first relied on the mouse colon cancer model MC38 where we found that intratumoral injection of cGAMP to tumor-bearing mice induced tumor growth control in a STING-dependent manner (online supplemental figure S2A).

**Figure 1:**
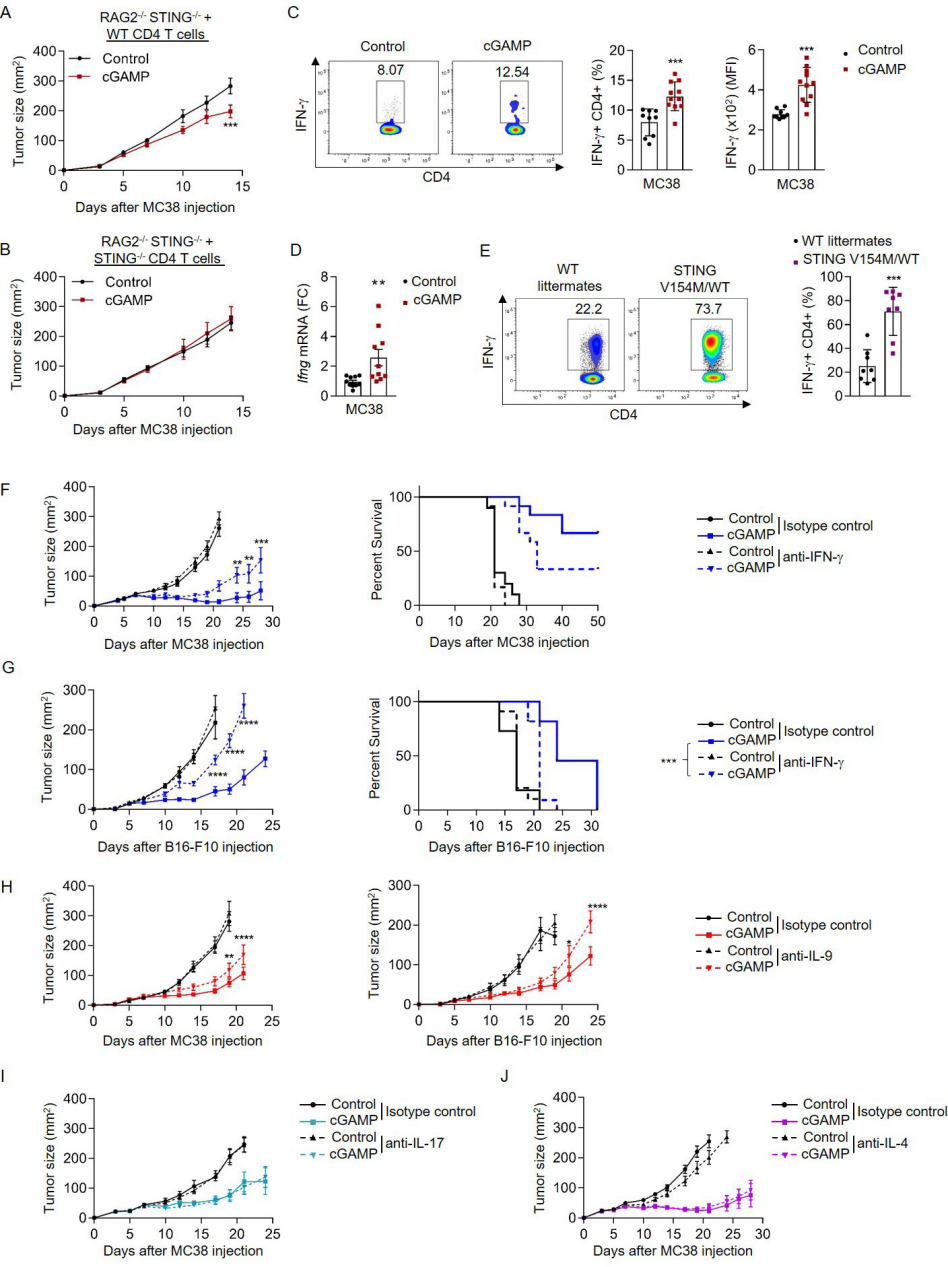
STING activation enhances CD4 T-cell effector functions in vivo. (A, B) MC38 tumor size in STING^−/−^ RAG2^−/−^ mice reconstituted with WT (A) or STING^−/−^ (B) CD4 T cells and STING^−/−^ CD8 T cells, and treated or not intratumorally with cGAMP. Mean±SEM of n=8–9 mice per group pooled from two independent experiments. (C, D) TIL analysis from MC38 tumor-bearing WT mice after intratumoral cGAMP treatment. (C) IFN-γ production (representative plots (left); frequency (middle) and MFI (right)). (D) *Ifng* mRNA expression (FC) from FACS-sorted CD4 T cells. Mean±SD from three independent experiments where each dot represents one mouse (n=9–11 mice). (E) IFN-γ production (representative plots (left) and frequency (right)) in splenic CD4 T cells isolated from STING V154M/WT mice or WT littermates. Mean±SD from three independent experiments where each dot represents one mouse (n=8 mice). (F, G) Tumor size (left) and survival (right) in MC38 (F) and B16-F10 (G) tumor-bearing mice treated or not intratumorally with cGAMP as well as intraperitoneally with anti-IFN-γ or its control IgG. Mean±SEM of n=10–12 (F) and n=11 (G) mice per group pooled from two independent experiments. (H) Tumor size in MC38 (left) and B16-F10 (right) tumor-bearing mice treated or not intratumorally with cGAMP as well as intraperitoneally with anti-IL-9 antibodies or its control IgG. Mean±SEM of n=11 (left) and n=8–10 (right) mice per group pooled from two independent experiments. (I, J) Tumor size in MC38 tumor-bearing mice treated or not intratumorally with cGAMP as well as intraperitoneally with anti-IL-17 (I) or anti-IL-4 (J) antibodies or their respective control IgG. Mean±SEM of n=11–12 (I) and n=10–11 (J) mice per group pooled from two independent experiments. P values (*p<0.05,**p<0.01, ***p<0.001, ****p<0.0001) determined by unpaired t-test (C–E), Two-way analysis of variance (A, B, F–J) or log-rank test (F, G, right; survival only). cGAMP, 2′3′-cyclic guanosine monophosphate–adenosine monophosphate; FC, fold change; IFN-γ, interferon gamma; IL, interleukin; MFI, mean fluorescence intensity; STING, stimulator of interferon genes; TIL, tumor-infiltrating lymphocyte; WT, wild type.

Analysis of effector cytokines *Ifng*, *Il9, Il17a* and *Il4* mRNA in tumor-infiltrating immune cells revealed that *Ifng* and *Il9*, but not *Il17a* or *Il4*, expression was increased following cGAMP administration (online supplemental figure S2B). We and others showed that CD4 T cells support the induction of CD8 T cell-dependent anticancer immune responses,^1 5 38^ and recent studies suggested that CD4 T cells could contribute to the antitumor properties of STING ligands.^39^ To test this, we have crossed Rag2^–/–^ mice to STING-deficient mice and reconstituted them with either WT or STING-deficient CD4 T cells as well as with STING-deficient CD8 T cells. We subsequently engrafted MC38 tumor cells to these mice and treated them with cGAMP (figure 1A, B). cGAMP treatment was only effective in mice reconstituted with WT CD4 T cells, indicating that CD4 T cells can be directly affected by STING agonists in vivo in the absence of STING-expressing APCs (figure 1A, B), further underscoring the essential contribution of CD4 T cells in the anticancer efficacy of cGAMP. We then analyzed tumor-infiltrating CD4 T cells and found an increased expression of interferon-beta (*Ifnb1*) and interferon-stimulated gene (ISG) transcripts (*Ccl5*, *Ifit1*, and *Mx2*) in cells isolated from mice treated with cGAMP compared with controls (online supplemental figure S2C). Interestingly, we also found that cGAMP increased IFN-γ production from tumor-infiltrating CD4 T cells (figure 1C, D) as well as from MC38-OVA tumor-infiltrating OVA-specific CD4 T cells (online supplemental figure S2D), suggesting that STING activation enhances CD4 T-cell effector functions in vivo. To test this, we evaluated the impact of the gain-of-function V154M mutation of STING, which drives its constitutive activation,^29^ on CD4 T cell-derived IFN-γ secretion. Effector CD4 T cells isolated from STING V154M/WT mouse spleens featured increased IFN-γ production on ex vivo restimulation than those from control littermates (figure 1E). Transcriptional analysis of CD4 T cells isolated from STING V154M/WT mice by RNAseq revealed an upregulation of T_H_1-related genes compared with control littermates (online supplemental figure S2E). We then tested the contribution of IFN-γ to the anticancer activity of cGAMP in vivo. In the immunogenic MC38 model, we found that IFN-γ neutralization impaired cGAMP antitumor effect as shown by the reduction of both cGAMP-mediated tumor growth control and mouse survival (figure 1F). When we performed a similar experiment in mice bearing poorly immunogenic B16-F10 tumors, we noted that the beneficial anticancer of cGAMP was abrogated by IFN-γ blockade (figure 1G). The latter observation is in line with the previously reported key contribution of IFN-γ in preventing B16 tumor growth in vivo.^40^ To further test whether IFN-γ is important for the anticancer effects of cGAMP mediated by CD4 T cells, we have repeated our adoptive transfer model experiments presented in figure 1A upon neutralization of IFN-γ. Rag2^−/−^STING^−/−^ mice reconstituted with WT CD4 T cells and injected with anti-IFN-γ antibody have a markedly reduced response to cGAMP compared with mice injected with isotype control, demonstrating that IFN-γ is a key contributor to the anticancer effects of cGAMP mediated by CD4 T cells in vivo (online supplemental figure S2F). IFN-γ neutralization does not fully abrogate the CD4 T cell-mediated anticancer effects of cGAMP in vivo, suggesting that other effector cytokines could also contribute to the anticancer effect of cGAMP. Because our analyses of tumor-bearing mice treated with cGAMP also revealed an increased frequency of IL-9-producing CD4 T cells in the tumor microenvironment (online supplemental figure S2G, H), we finally evaluated the contribution of other CD4 T cell-derived cytokines (IL-9, IL-17, and IL-4) to the anticancer effects of cGAMP. While IL-9-neutralizing antibodies reduced the anticancer activity of cGAMP in both MC38 and B16-F10 tumor models (figure 1H), neutralization of IL-4 and IL-17 had no effect on cGAMP-mediated tumor growth control (figure 1I, J). Overall, these results indicate that STING activation enhances CD4 T-cell effector functions and reveal that IFN-γ and IL-9 contribute to the antitumor activity of cGAMP in vivo.

### STING ligands enhance T_H_1 and T_H_9 cell differentiation in vitro

**Figure 2:**
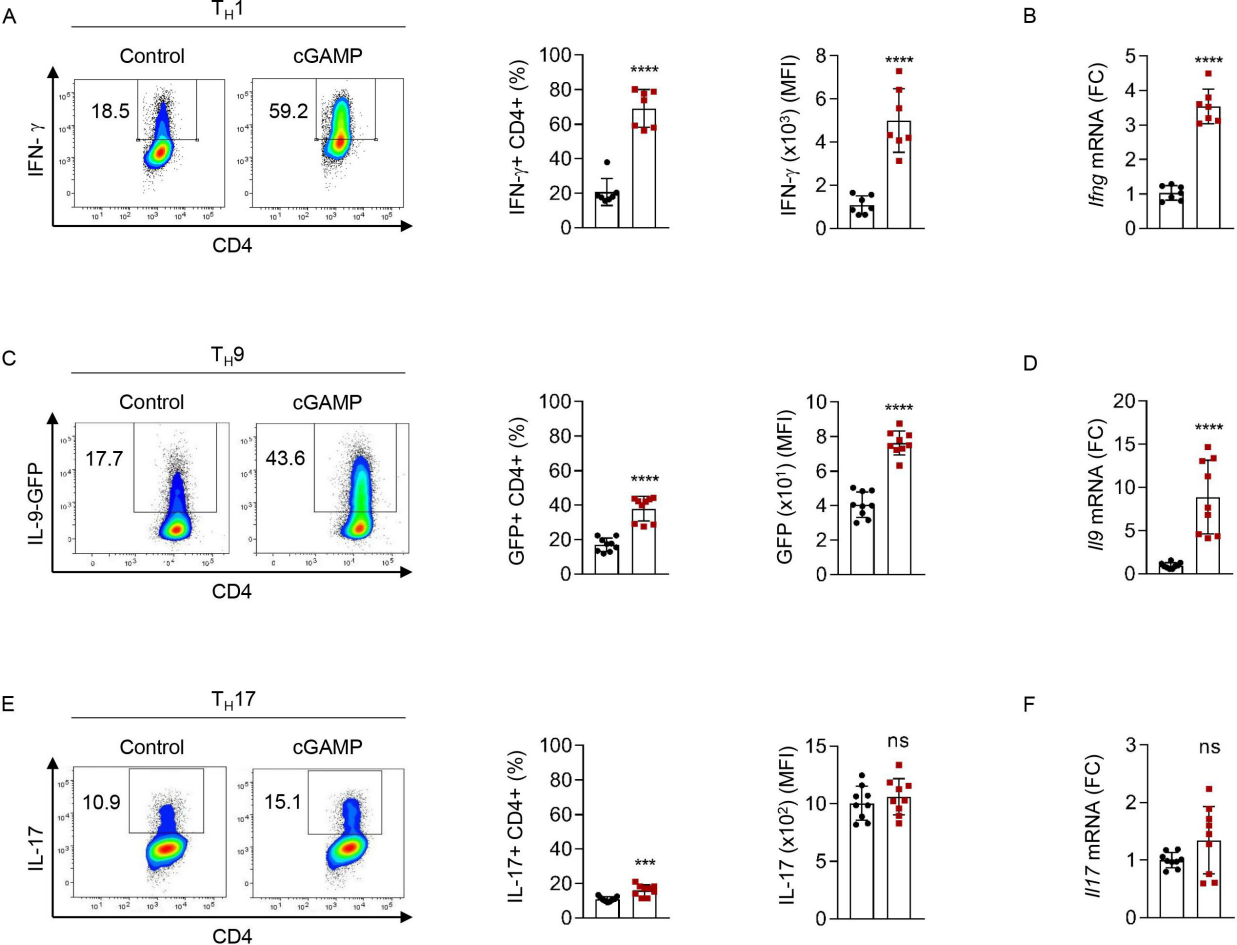
cGAMP enhances T_H_1 and T_H_9 cell differentiation in vitro. (A, B) IFN-γ production (representative plots (A, left); frequency (A, middle) and MFI (A, right) and mRNA expression (FC), (B) from WT naive CD4 T cells stimulated with cGAMP and polarized into T_H_1 cells. (C, D) IL-9 production (representative plots (C, left); frequency (C, middle) and MFI (C, right) and mRNA expression (FC), (D) from IL-9-GFP naive CD4 T cells stimulated with cGAMP and polarized into T_H_9 cells. (E, F) IL-17 production (representative plots (E, left); frequency (E, middle) and MFI (E, middle) and mRNA expression (FC) (F) from WT naive CD4 T cells stimulated with cGAMP and polarized into T_H_17 cells. Mean±SD of replicates pooled from three independent experiments. P values (***p<0.001, ****p<0.0001) determined by unpaired t-tests. cGAMP, 2′3′-cyclic guanosine monophosphate–adenosine monophosphate; FC, fold change; GFP, green fluorescent protein; IFN-γ, interferon gamma; IL, interleukin; MFI, mean fluorescence intensity; ns, not significant.

**Figure 3:**
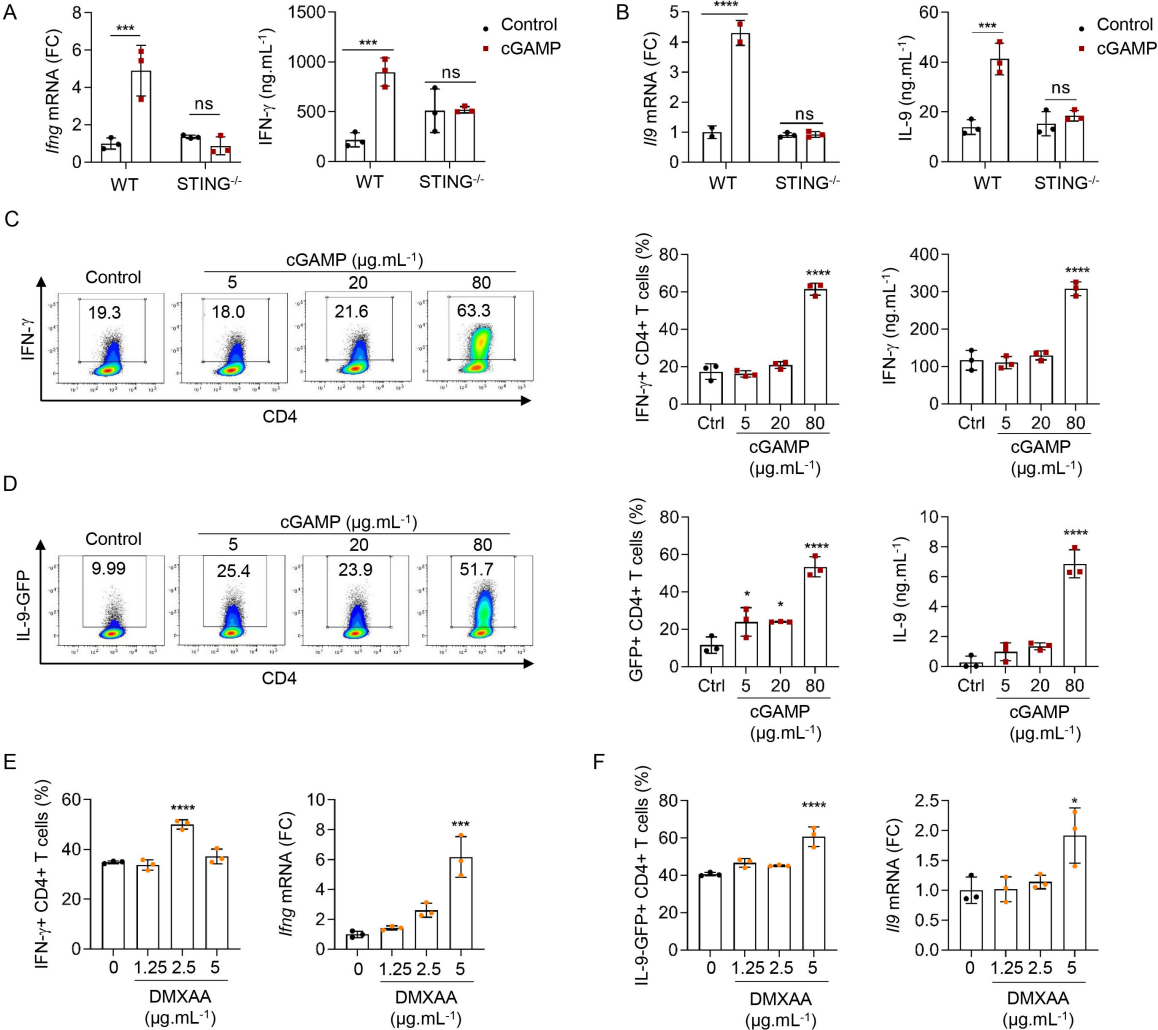
STING ligands enhance T_H_1 and T_H_9 cell differentiation in vitro. (A, B) *Ifng* (A) or *Il9* (B) mRNA expression (FC, left) and IFN-γ (A) or IL-9 (B) secretion (right) from WT or STING^−/−^ naive CD4 T cells stimulated with cGAMP or control and polarized into T_H_1 (A) or T_H_9 (B) cells. (C, D) IFN-γ (C) or IL-9 (D) production (representative plots (left), frequency (middle)) and secretion (right) from WT (C) or IL-9-GFP (D) naive CD4 T cells stimulated with cGAMP or control and polarized into T_H_1 (C) or T_H_9 (D) cells. (E, F) IFN-γ (E) or IL-9 (F) production (frequency, left) and *Ifng* (A) or *Il9* (B) mRNA expression (FC, right) from WT (E) or IL-9GFP (F) naive CD4 T cells stimulated with DMXAA or dimethyl sulfoxide (DMSO) and polarized into T_H_1 (E) or T_H_9 (F) cells. Mean±SD of replicates from one experiment representative of three (C, D) or two (A, B, E, F) experiments. P values (*p<0.05, ***p<0.001, ****p<0.0001) determined by two-way ANOVA (A, B) or one-way ANOVA (C–F). ANOVA, analysis of variance; cGAMP, 2′3′-cyclic guanosine monophosphate–adenosine monophosphate; FC, fold change; GFP, green fluorescent protein; IFN-γ, interferon gamma; IL, interleukin; ns, not significant; WT, wild type.

Because of the key contribution of IFN-γ and IL-9 to the anticancer effects induced by cGAMP in vivo, we next assessed whether STING signaling directly affects the differentiation of CD4 T cells in vitro. For this, we tested if cGAMP or negative control dinucleotide (control), which does not activate STING signaling, could modulate the polarization of naive CD4 T cells into T_H_1, T_H_9 or T_H_17 cells in the absence of APCs. In line with our in vivo data, naive CD4 T cells activated with anti-CD3 and anti-CD28 antibodies in the presence of cGAMP, and polarized into T_H_1 and T_H_9 cells, respectively, featured enhanced IFN-γ and IL-9 expression and secretion 3 days after differentiation initiation compared with control (figure 2A–D). In contrast, we noted that cGAMP featured limited and variable effects on T_H_17 cell differentiation (figure 2E, F). We found that the ability of cGAMP to enhance T_H_1 and T_H_9 cell differentiation was lost when CD4 T cells were isolated from STING-deficient mice (STING^−/−^), underscoring the STING-dependent activity of cGAMP in CD4 T cells (figure 3A, B).

We also tested the ability of increasing doses of cGAMP to affect T_H_1 and T_H_9 cell differentiation. Increasing doses of cGAMP enhanced T_H_1 and T_H_9 cell differentiation without significantly affecting cell death at the doses tested (figure 3C, D, and online supplemental figure S3A, B). The mouse STING ligand DMXAA, which is cell-permeable and potently activates STING, was proposed to induce T-cell death when used at the dose of 10 µg/mL.^26^ Here, we found that while low doses of DMXAA enhanced T_H_1 differentiation, higher doses began to concomitantly trigger cell death (figure 3E and online supplemental figure S3C). A higher dose of DMXAA was required to observe enhanced T_H_9 differentiation (figure 3F) in line with the fact that T_H_9 cells appeared less sensitive to DMXAA-induced cell death than T_H_1 cells (online supplemental figure S3D). While these results are in agreement with previous studies proposing that potent STING activation in T cells triggers cell death,^24 26^ they also indicate that milder STING activation can enhance T-cell effector properties without killing them, a finding that will be of importance as this pathway is targeted for cancer therapy development. Interestingly, we noted a higher sensitivity of T_H_1 cells to STING-induced death (online supplemental figure S3A–D, right panels), suggesting that the balance between enhancement of effector properties and cell death varies between CD4 T-cell subsets. Altogether, these data show that the strength of STING engagement defines the consequences of STING activation on CD4 T-cell fate.

Overall, our results indicate that ligand-induced STING activation enhances the secretion of effector cytokines IFN-γ and IL-9 from T_H_1 and T_H_9 cells and thus controls the effector functions of both CD4 T-cell subsets.

### Transcriptional regulation of T_H_1 and T_H_9 cell differentiation following STING activation

**Figure 4:**
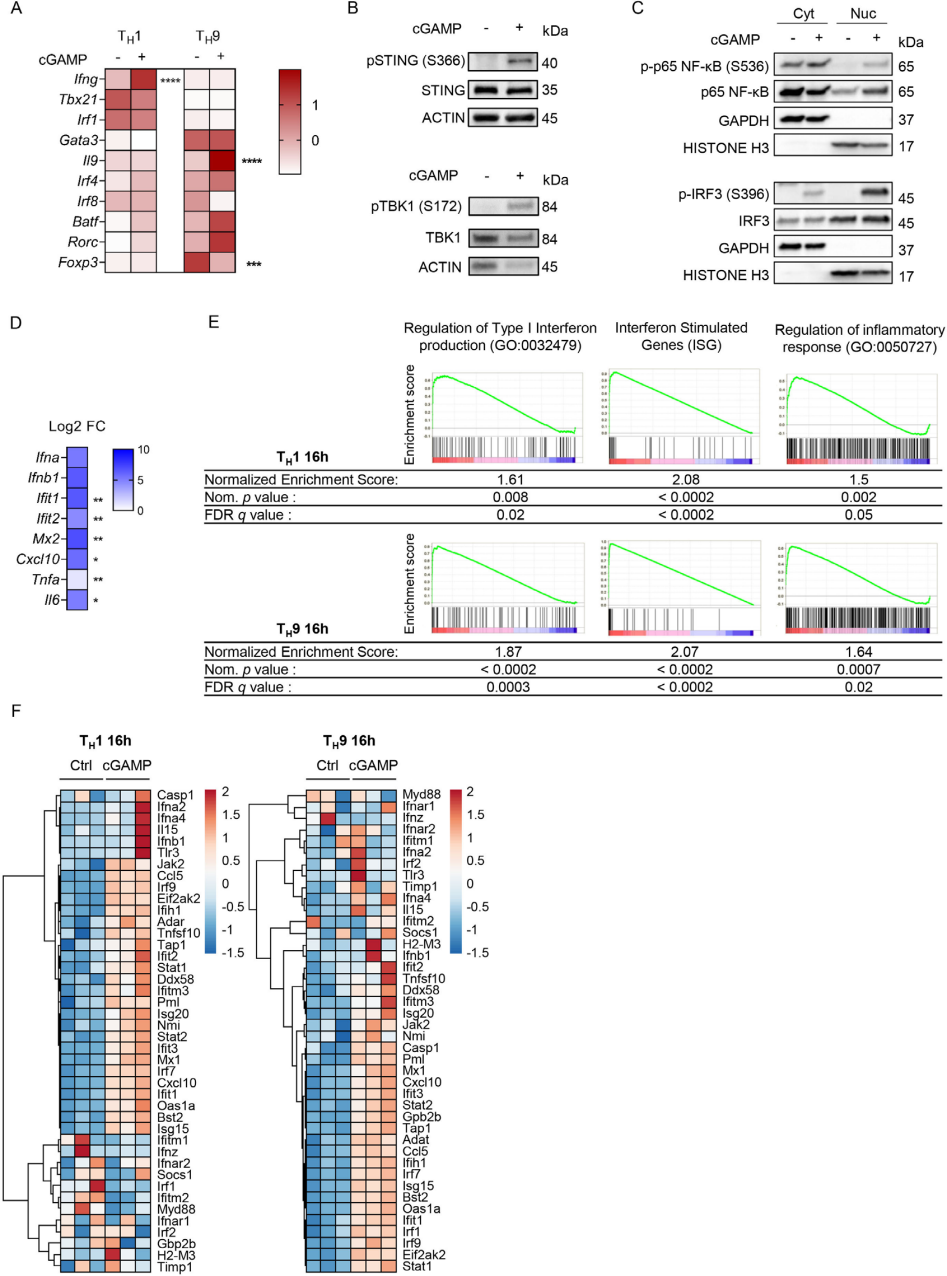
Transcriptional regulation of T_H_1 and T_H_9 cell differentiation following STING activation. (A) Heatmap representing *Ifng*, *Tbx21*, *Irf1*, *Gata3*, *Il9*,*Irf4*, *Irf8*, *Batf*, *Rorc*, and *Foxp3* mRNA expression (Z-score) from naive CD4 T cells stimulated with cGAMP or control and polarized into T_H_1 or T_H_9 cells for 72 hours. (B–D) WT naive CD4 T cells stimulated with cGAMP or control for 4(B) or 6(C, D) hours. (B) STING, p-STING, TBK1 and p-TBK1 protein levels. (C) Cyt and Nuc localization of IRF3, p65 NF-κB and their phosphorylated forms. (D) Heatmap representing *Ifna*, *Ifnb1*, *Il6*, *Tnfa*, *Ifit1*, *Ifit2*, *Mx2* and *Cxcl10* mRNA expression (Log2FC) between CD4 T cells stimulated with cGAMP and control. Mean of replicates from three independent experiments. P values (*p<0.05, **p<0.01, ***p<0.001, ****p<0.0001) determined by two-way analysis of variance (A) or unpaired t test (D). (E, F) RNA sequencing analysis from WT naive CD4 T cells stimulated with cGAMP or control and polarized into T_H_1 or T_H_9 cells for 16 hours. Biological replicates from three independent experiments. (E) Gene set enrichment analysis comparing expression in cGAMP-stimulated cells to control cells. Enrichment plot and score, Nom. P value and FDR *q* value shown for the three gene sets. (F) Heatmaps illustrating the hierarchical clustering of expression levels (rld values) of ISGs. cGAMP, 2′3′-cyclic guanosine monophosphate–adenosine monophosphate; Cyt, cytosolic; FDR, false discovery rate; ISG, interferon-stimulated gene; Nom, nominal; Nuc, nuclear; STING, stimulator of interferon genes; WT, wild type.

We evaluated whether STING activation affected T_H_1 and T_H_9 cell differentiation at the transcriptional level. While the expression levels of *Ifng* and *Il9* were strongly enhanced in differentiating T_H_1 and T_H_9 cells on cGAMP treatment, no notable change in the expression of the T_H_1-specific and T_H_9-specific transcription factors *Tbx21/Irf1* and *Spi1/Irf4/Irf8/Batf*, respectively, was detected (figure 4A and online supplemental figure S4A). In line with their enhanced expression of *Il9*, T_H_9 cells treated with cGAMP featured lower expression levels of *Foxp3*^7^ (figure 4A). No significant induction of *Gata3* or *Rorc* expression in T_H_1 and T_H_9 cells on cGAMP treatment was found, suggesting that cGAMP enhances but does not skew T_H_1 and T_H_9 cell differentiation (figure 4A).

To unravel the mechanisms explaining how STING activation promotes enhanced T_H_1 and T_H_9 cell differentiation, we interrogated the intracellular events following activation of STING with cGAMP in mouse CD4 T cells. We found that cGAMP triggered STING and TBK1 phosphorylation as well as Nuc translocation of the phosphorylated forms of p65 and IRF3 (figure 4B, C, and online supplemental figure S4B, C), which are features of STING activation.^41^ In addition, type I IFN (*Ifna* and *Ifnb1*), ISG (*Ifit1*, *Ifit2*, *Mx2* and *Cxcl10*) and proinflammatory gene (*Tnfa* and *Il6*) transcript levels measured in CD4 T cells 6 hours after stimulation with cGAMP were increased (figure 4D and online supplemental figure S4D).

We next performed RNAseq analysis on T_H_1 and T_H_9 differentiated for 16 and 48 hours after stimulation with cGAMP or control. Because we noted cGAMP induced type I IFN and ISG expression in CD4 T cells in vivo (online supplemental figure S2C), we investigated whether cGAMP was intrinsically triggering a global transcriptional program in differentiating T_H_1 and T_H_9 cells that was linked with the induction of a typical type I IFN-driven response. For this, we performed GSEA for type I IFN production (GO:0032479), ISGs and regulation of inflammatory response signatures (GO:0050727). GSEA revealed that these three gene sets were enriched in both T_H_1 and T_H_9 cells stimulated with cGAMP 16 and 48 hours after in vitro polarization (figure 4E and online supplemental figure S4E). Finally, *Trim30a*, which was recently identified as a negative feedback regulator of the STING pathway in DCs,^42^ and *Usp18*, which was shown to be involved in STING deubiquitination and stabilization,^43^ were strongly induced in both T_H_1 and T_H_9 cells stimulated with cGAMP (online supplemental table S2). Gene transcript analyses also revealed that cGAMP triggers a transcriptional program typically related to STING activation, which notably leads to the expression of type I IFN and ISGs. Importantly, this program is maintained over time as reflected by the expression of ISG including *Isg15*, *Mx1*, *Mx2*, *Cxcl10*, and *Irf7* that was markedly increased in CD4 T cells stimulated with cGAMP and differentiated into T_H_1 or T_H_9 cells for 16 or 48 hours (figure 4F and online supplemental figure S4F). Finally, in line with our previous results, T_H_1 and T_H_9 polarized after cGAMP stimulation featured a marked increase in *Ifng* and *Il9* expression, respectively, but also in other cytokines/chemokines including *Gzmb*, *Il2* and *Ccl4* for T_H_1 cells and *Il21*, *Tnfsf4* (OX40L), *Tnfsf8*, as well as *Il10* for T_H_9 cells (online supplemental figure S4G, H). These analyses were confirmed by our investigation of the biological pathways engaged in these cells using EnrichR (online supplemental figure S4I).

Collectively, these data reveal that the engagement of STING in differentiating T_H_1 and T_H_9 cells not only enhances the expression of their respective effector cytokines but also engages a transcriptional program driving the expression of type I IFNs and proinflammatory genes.

### STING signaling enhances T_H_1 cell differentiation through IRF3 signaling

**Figure 5:**
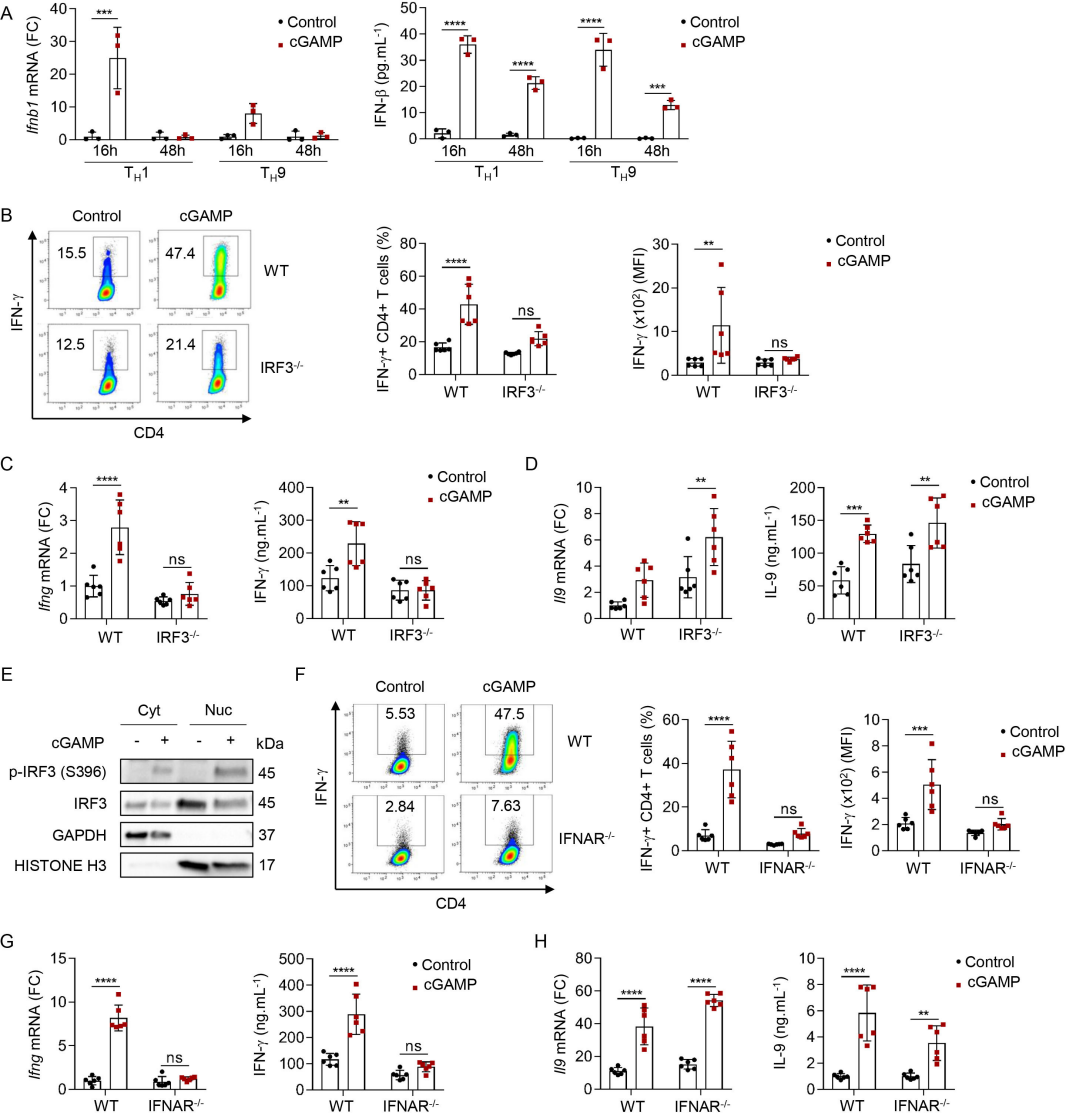
STING signaling enhances T_H_1 cell differentiation through IRF3 signaling. (A) *Ifnb1* mRNA expression (FC, left) and IFN-β secretion (right) from WT naive CD4 T cells stimulated with cGAMP or control and polarized into T_H_1 and T_H_9 for 16 and 48 hours. Mean±SD of replicates from one experiment representative of two independent experiments. (B–D) IFN-γ production (representative plots (B, left), frequency (B, middle), and MFI (B right)), *Ifng* (C) or *Il9* (D) mRNA expression (FC, left) and IFN-γ (C) or IL-9 (D) secretion (right) from WT or IRF3^−/−^ naive CD4 T cells stimulated with cGAMP or control and polarized into T_H_1 (B, C) or T_H_9 (D) cells. (E) Cyt and Nuc localization of IRF3 and its phosphorylated form in WT naive CD4 T cells stimulated with cGAMP or control and polarized into T_H_1 for 16 hours. (F–H) IFN-γ production (representative plots (F, left), frequency (F, middle), and MFI (F, right)), *Ifng* (G) or *Il9* (H) mRNA expression (FC, left) and IFN-γ (G) or IL-9 (H) secretion (right) from WT or IFNAR^−/−^ naive CD4 T cells stimulated with cGAMP or control and polarized into T_H_1 (F, G) or T_H_9 (H) cells. Mean±SD of replicates pooled from two independent experiments (B–D, F–H). P values (**p<0.01, ***p<0.001, ****p<0.0001) determined by two-way analysis of variance. cGAMP, 2′3′-cyclic guanosine monophosphate–adenosine monophosphate; Cyt, cytosolic; FC, fold change; IFN-γ, interferon gamma; IL, interleukin; ns, not significant; MFI, mean fluorescence intensity; Nuc, nuclear; STING, stimulator of interferon genes; WT, wild type.

Since we noted a strong induction of a type I IFN transcriptional program in T_H_1 and T_H_9 cells on STING activation, we next tested its relevance in the differentiation of these cells. First, we observed that cGAMP induced not only *Ifnb1* transcription but also the secretion of IFN-β from differentiating T_H_1 and T_H_9 cells (figure 5A). The induction of type I IFN expression in response to STING activation in myeloid cells has been shown to depend on the transcription factor IRF3.^44 45^ We thus tested the contribution of IRF3 in mediating the effects of cGAMP in differentiating T_H_1 and T_H_9 cells. For this, we polarized naive CD4 T cells from control and *Irf3*-deficient (IRF3^−/−^) mice into T_H_1 and T_H_9 cells. IRF3 contributed to the cGAMP-induced IFN-γ production from differentiating T_H_1 cells (figure 5B, C). However, cGAMP conserved its ability to enhance the differentiation of IRF3-deficient naive T cells into T_H_9 cells (figure 5D). In line with this role for IRF3 in cGAMP-driven T_H_1 differentiation, levels of phosphorylated IRF3 remained increased in the cytosol and the nucleus of T_H_1 cells 16 hours after differentiation initiation (figure 5E and online supplemental figure S5A). We then directly assessed the functional consequences of cGAMP-driven type I IFN secretion in differentiating T_H_1 and T_H_9 cells using type I IFN receptor-deficient mice (IFNAR^−/−^). We found that T_H_1 but not T_H_9 cells relied on cGAMP-induced type I IFN receptor-dependent for the enhancement of their differentiation (figure 5F–H). Overall, these results underscore a key contribution of STING-induced IRF3 activation and type I IFN secretion in the cGAMP-driven enhancement of T_H_1 cell differentiation.

### STING signaling enhances T_H_9 effector functions through activation of mTOR signaling

**Figure 6:**
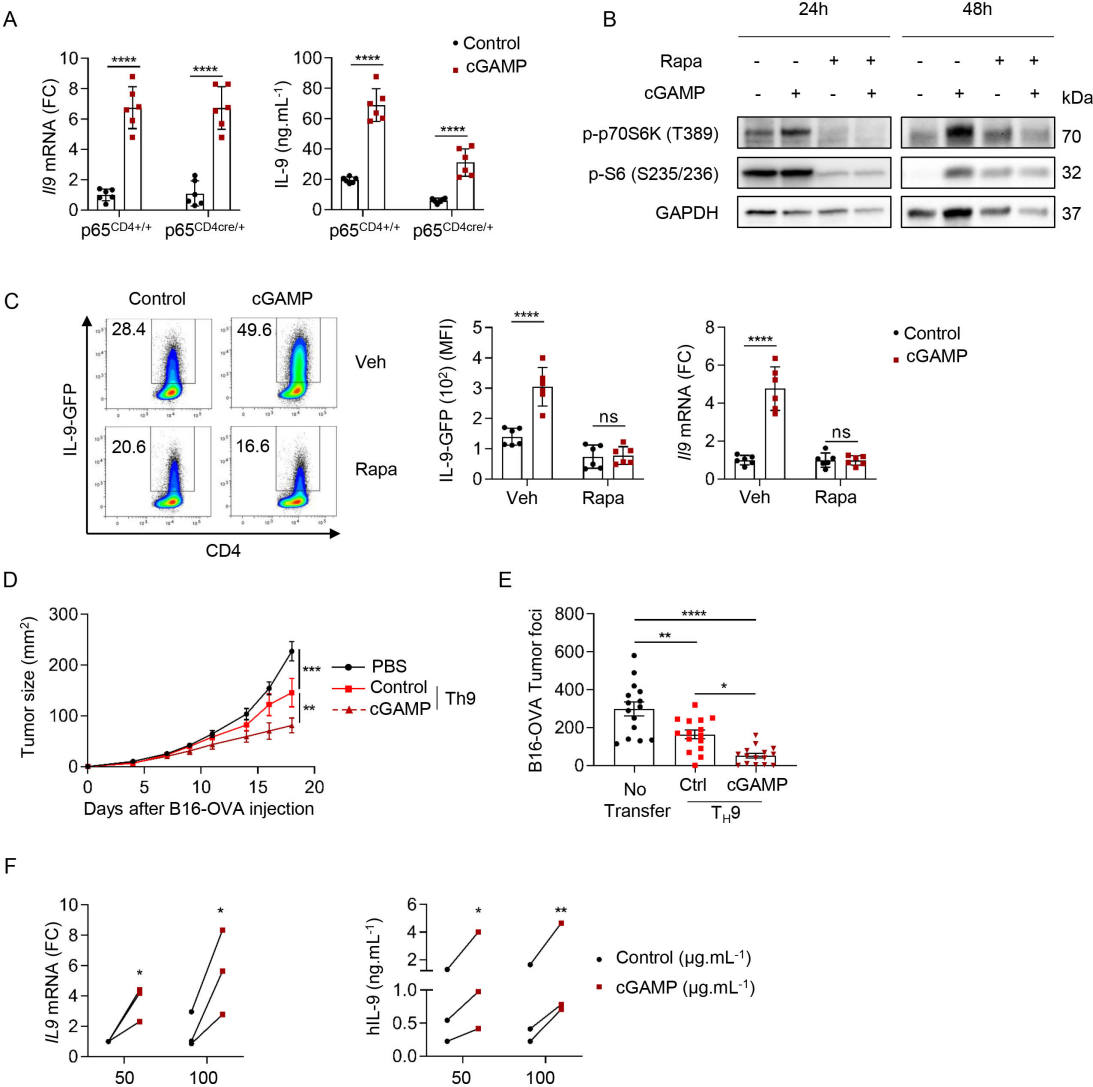
STING signaling enhances T_H_9 effector functions through activation of mTOR signaling. (A) *Il9* mRNA expression (FC, left) and IL-9 secretion (right) from p65^CD4+/+^ or p65^CD4Cre/+^ naive CD4 T cells stimulated with cGAMP or Ctrl and polarized into T_H_9 cells. Mean±SD of replicates pooled from two independent experiments. (B, C) WT (B) or IL-9-GFP (C) naive CD4 T cells stimulated with cGAMP or Ctrl in the presence of Rapa (10 nM) or Veh and then polarized into T_H_9 cells for 24 hours and 48 hours (B) or 3 days (C). (B) Levels of phosphorylated p70S6K (P-p70S6K) and S6 (P–S6). (C) IL-9 production (representative plots, left; MFI, middle; and *Il9* mRNA expression (FC), right). Mean±SD of replicates pooled from two independent experiments. (D) Tumor size in B16-OVA tumor-bearing mice injected intravenously with PBS (Mock), OT-II T_H_9 cells or cGAMP-stimulated OT-II T_H_9 cells 5 days after tumor cell inoculation. (E) Tumor foci enumerated 14 days after intravenous injection of B16-OVA tumor cells and intravenous injection of PBS (no transfer), OT-II T_H_9 cells or cGAMP-stimulated OT-II T_H_9 cells 1 day after tumor cell inoculation. Mean±SEM of n=11–12 (D) and n=15 (E) mice per group pooled from two independent experiments. (F) *IL9* mRNA expression (FC) (left) and hIL-9 secretion (right) from human naive CD4 T cells stimulated with cGAMP or Ctrl and polarized into T_H_9 cells. Mean of replicates from three independent experiments and each dot represents one donor. P values (*p<0.05, **p<0.01, ***p<0.001, ****p<0.0001) determined by two-way ANOVA (A, C, D, F,) or one-way ANOVA (E). ANOVA, analysis of variance; cGAMP, 2′3′-cyclic guanosine monophosphate–AMP; Ctrl, control; FC, fold change; IL, interleukin; ns, not significant; Rapa, rapamycin; STING, stimulator of interferon genes; Veh, vehicle.

STING engages NF-κB signaling and the activation of NF-κB-p65 by STING is essential for antiviral immunity.^46^ NF-κB (p65) was also proposed to bind the mouse *Il9* promoters^47^ and enhance *Il9* expression in the context of T_H_9 cell differentiation with proinflammatory factors.^9^ We generated mice conditionally lacking p65 expression in T cells (p65^CD4cre/+^) to investigate the ability of STING activation to affect T_H_9 cell differentiation in the absence of p65. While p65 deficiency reduced IL-9 secretion from differentiating T_H_9 cells, it failed to prevent the ability of cGAMP to enhance T_H_9 cell differentiation (figure 6A). mTOR activation is required for STING-mediated IFN-β production in T cells.^48^ Because activation of mTOR signaling has been reported to be required for T_H_9 cell differentiation,^49^ we tested whether cGAMP could affect mTOR signaling in differentiating T_H_9 cells. We found that cGAMP induced the phosphorylation of the downstream effectors of mTOR pathway P70S6 kinase and S6 ribosomal protein 48 hours after T_H_9 differentiation initiation and that these phosphorylation events were compromised by the addition of the mTOR inhibitor Rapamycin, suggesting that cGAMP activates mTOR signaling in differentiating T_H_9 cells (figure 6B and online supplemental figure S5B). In addition, we found that Rapamycin abrogated the effect of cGAMP on *Il9* transcription as well as protein expression and secretion (figure 6C and online supplemental figure S5C). Overall, these results suggest that the ability of cGAMP to enhance the differentiation of T_H_9 cells relies on the activation of mTOR signaling.

T_H_9 cells have recently been proposed to harbor superior anticancer properties compared with T_H_1, T_H_2 or T_H_17 in vivo in the B16 melanoma tumor model.^4 6^ Given that our results showed that STING ligands enhance T_H_9 cell differentiation, we examined the ability of naive CD4 T cells stimulated with control or cGAMP and differentiated into T_H_9 cells to prevent cancer tumor growth in vivo. For this, B16-OVA tumor cells (ovalbumin-transfected B16-F10 melanoma cells) were first injected subcutaneously into mice. Five days after tumor cell engraftment, mice received an intravenous injection of T_H_9 cells polarized in vitro and derived from OT-II mice, which have a TCR specific for the ovalbumin peptide (323–339) presented by MHC class II molecules. As expected, cGAMP enhanced CD4 T cell-derived *Il9* expression from OT-II T_H_9 cells in vitro as compared with control (online supplemental figure S5D). Tumor growth monitoring revealed that cGAMP enhances the antitumor properties of T_H_9 cells upon adoptive transfer (figure 6D). We obtained similar results when B16-OVA cells were injected intravenously, as shown by the reduced number of B16-OVA lung tumor foci in mice which received OT-II T_H_9 cells stimulated with cGAMP compared with control T_H_9 cells (figure 6E). Finally, we also tested the anticancer potential of cGAMP-treated T_H_9 cells against B16F-10 cancer cells in vivo using Trp1 transgenic T cells, which recognize tyrosinase-related protein 1 a specific melanoma tumor antigen.^50^ We also found in that setting that cGAMP endowed antigen-specific T_H_9 cells with higher IL-9 production and superior anticancer functions (online supplemental figure S5E, F).

To investigate whether our findings on mouse CD4 T cells are relevant in a human setting, we obtained human blood samples from healthy volunteers, isolated human naive CD4 T cells and differentiated them into T_H_1 or T_H_9 cells with IL-12 or TGF-β and IL-4, respectively, after stimulation with cGAMP or negative control dinucleotide (control). In line with our results obtained with mouse T cells, we found that cGAMP promoted human T_H_1 and T_H_9 cell differentiation (figure 6F and online supplemental figure S5G). Altogether, these results show that the activation of STING signaling with cGAMP not only affects CD4 T-cell biology through the activation of innate immune responses but also cell intrinsically shapes both mouse and human T_H_1 and T_H_9 cell differentiation.

## Discussion

The contribution of CD4 T-cell effector responses to the antitumor immune effects induced by STING ligand administration in the tumor environment was elusive. Here, our results underscore a key role for IFN-γ and IL-9 in mediating the anticancer effects following STING ligand administration in vivo. We further show that cell-intrinsic STING activation enhances the effector and antitumor functions of T_H_1 and T_H_9 cells through two distinct molecular mechanisms, the engagement of the IRF3 and mTOR signaling, respectively. These findings uncover STING as an attractive target to improve CD4 T cell-mediated cancer immunotherapy.

STING was initially characterized to be essential for induction of antiviral immunity, notably because of its ability to promote the secretion of type I IFNs and proinflammatory mediators from DCs and macrophages.^16^ STING-driven activation of the innate immune system promotes adaptive immune responses that not only favor host defense against infections but also can drive the elimination of cancer cells,^45^ indicating the key importance of this molecular pathway for the maintenance of host homeostasis. While the central importance of STING is known to rely on its ability to bridge innate and adaptive immunity on activation, accumulating evidence suggests that adaptive immune cells can directly respond to STING engagement. B cell-intrinsic STING signaling was indeed proposed to promote antibody responses independently of type I IFNs.^51^ Furthermore, STING triggering can drive type I IFN responses in T cells.^48 52^ Analysis of knock-in mice STING N153S where STING signaling is constitutively active also revealed that T cells featured activation of mTOR and an activated phenotype, possibly resulting from a cell-intrinsic effect of the STING mutation.^53^ Whereas initial studies showed that STING activation induces T cell death, other investigations suggested that the strength of STING signaling differentially affected T-cell activation in a TCR-dependent manner.^48^ STING activation was also recently proposed to enhance the fitness of CD8 T cells, thereby contributing to anticancer immunity.^54^ How STING regulates the differentiation outcome of CD4 T cells, however, was incompletely understood. We find that the nature, potency and dose of STING ligands critically determine the fate of CD4 T cells after STING activation. We also demonstrate that CD4 T-cell polarizing conditions influence the consequences of cell-intrinsic STING activation, as illustrated by a reduced sensitivity to STING-induced cell death of T_H_9 cells compared with T_H_1 cells. This might be due to the control of T_H_1 cell-IFN-γ secretion through IRF3/IFN-β/IFNAR axis, which may also affect proliferation and death.^24 48^ Although prolonged STING stimulation was proposed to downregulate mTOR signaling in T_H_0 cells,^48^ we instead noted that STING activation stimulated mTOR signaling along with IL-9 production in T_H_9 cells. While this is in line with previous observations that mTOR signaling harnesses T_H_9 cell functions through enhanced glycolytic activity,^55^ this also shows that signaling and outcome of STING activation can be distinct in different T-cell subsets. Our results overall indicate that STING signaling directly shapes the differentiation of T_H_1 and T_H_9 cells, thereby lending further support to a determinant function of cell-intrinsic STING signaling in T-cell fate. Whether STING activation additionally affects T-cell persistence in vivo warrants further investigations.

T_H_9 cells have emerged as a unique CD4 T-cell subset of particular interest for adoptive cell therapy of cancer. Compared with other CD4 T-cell subsets, murine T_H_9 cells elicit the highest antitumor response upon adoptive transfer because of their superior persistence, cytolytic functions and resistance to T-cell dysfunction.^4^ Importantly, human chimeric antigenic receptor (CAR) T cells cultured under T_H_9-polarizing conditions exert enhanced antitumor activity compared with cells cultured with IL-2, underscoring the clinical relevance of T_H_9 cells for cancer therapy.^56^ Previous studies reported that cell-extrinsic signals could favor T_H_9 cell differentiation. Indeed, proinflammatory factors secreted by APCs as well as engagement of costimulatory molecules like OX40 were shown to favor T_H_9 cell differentiation.^12 57^ Here, we extend these findings by identifying how T_H_9 cell differentiation can be regulated cell intrinsically following the triggering of the STING signaling pathway, which favors the promotion of inflammation. Our results show not only that CD4 T cell-intrinsic STING activation potentiates T_H_9 cell antitumor effect upon adoptive transfer in mice but also that human T_H_9 cell differentiation can be enhanced through STING activation. While STING agonists were recently suggested to synergize with CAR T cells and enhance their ability to control tumor growth,^58^ our findings provide impetus for studying the relevance of STING activation in T_H_9 cells in the context of adoptive T-cell therapy for cancer.

The ability of STING to regulate adaptive immunity is actually reminiscent of the cell-intrinsic activity of toll-like receptors (TLRs) in CD4 T cells. Indeed, while TLR activation was initially solely considered to bridge innate and adaptive immunity, it is now clear that TLR can also directly affect CD4 T-cell responses.^59 60^ Importantly, synthetic CDNs, nanoparticulate STING agonists, as well as small-molecule STING inhibitors have recently been shown to exhibit functional activities in vivo.^61–63^ Our findings could thus be therapeutically exploited to manipulate CD4 T-cell responses in infections, inflammatory diseases, and cancers.

## Data Availability

Data are available upon reasonable request. RNAseq data are deposited in the Gene Expression Omnibus database under accession number GSE147300.
