# Supplementary material for: CD4 T cell-intrinsic STING signaling controls the differentiation and effector functions of TH1 and TH9 cells
Source: J Immunother Cancer. 2022 Jan 28;10(1):e003459. doi: 10.1136/jitc-2021-003459 (PMC8804688; doi:10.1136/jitc-2021-003459)
Supplement: Supplementary data [file jitc-2021-003459supp001.pdf]

Supplementary Materials

Figure S1: Gating strategies

Figure S2: The antitumor effect of cGAMP *in vivo* requires STING and modulates CD4 T cell effector functions

Figure S3: STING ligands enhance the production of effector cytokines from *in vitro*-polarized T_H1 and T_H9 cells rather than affecting cell viability

Figure S4: STING signaling is activated in CD4 T cells and maintained after 48h of polarization.

Figure S5: STING-driven enhancement of T_H1 and T_H9 cell differentiation respectively involves IRF3 and mTOR signaling

Table S1. Primers and TaqMan assays used for mouse and human gene expression quantification

Table S2: Genes most differentially expressed in cGAMP-stimulated T_H1 and T_H9 cells

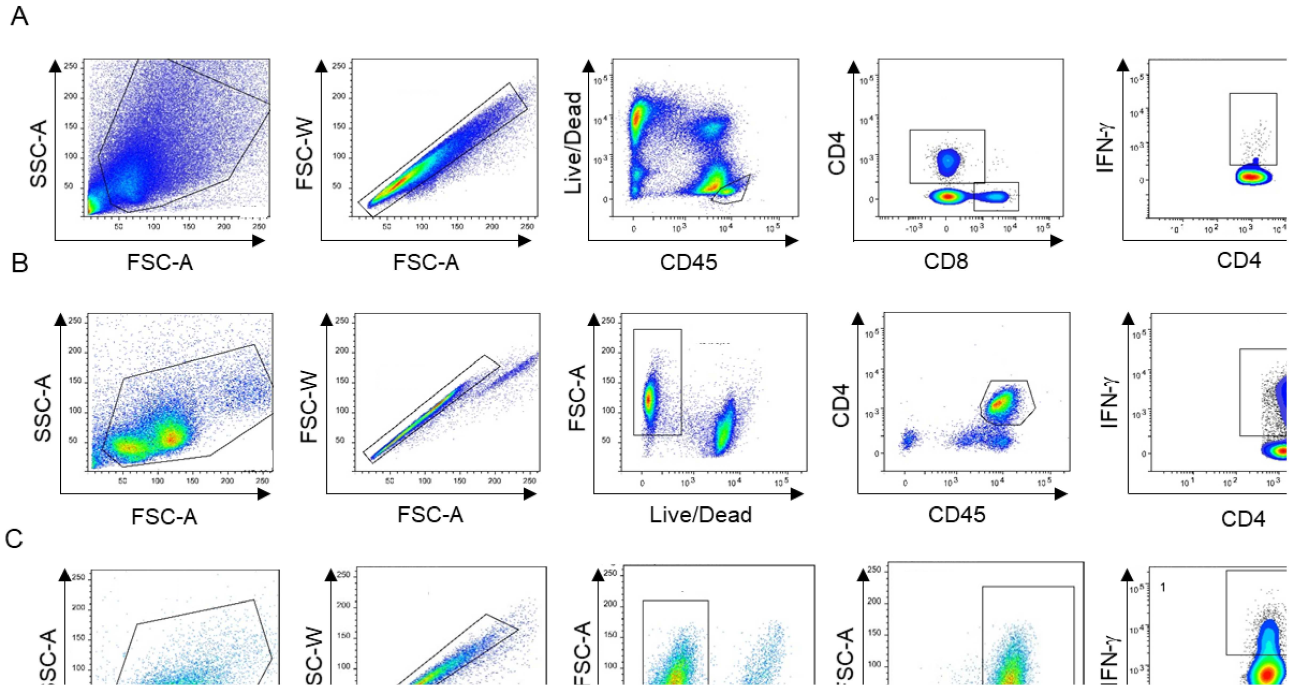

Figure S1: Gating Strategies

Figure S1: Gating Strategies

(A) Gating strategies for IFN-γ^+ or IL-9^+ CD4 T cell identification in tumor infiltrating cells.

(B) Gating strategies for IFN-γ^+ CD4 T cell identification in splenocytes from STING V154M/WT mice or WT littermates.

(C-E) Gating strategies for IFN-γ^+ **(C)**, GFP$^+$ **(D)**, IL-17^+ **(E)** CD4 T cell identification after T_H1 **(C)**, T_H9 **(D),** and T_H17 **(E)** differentiation *in vitro*.

Benoit-Lizon I, *et al. J Immunother Cancer* 2022; 10:e003459. doi: 10.1136/jitc-2021-003459

Figure S2: The antitumor effect of cGAMP in vivo requires STING and modulates CD4 T cell effector functions

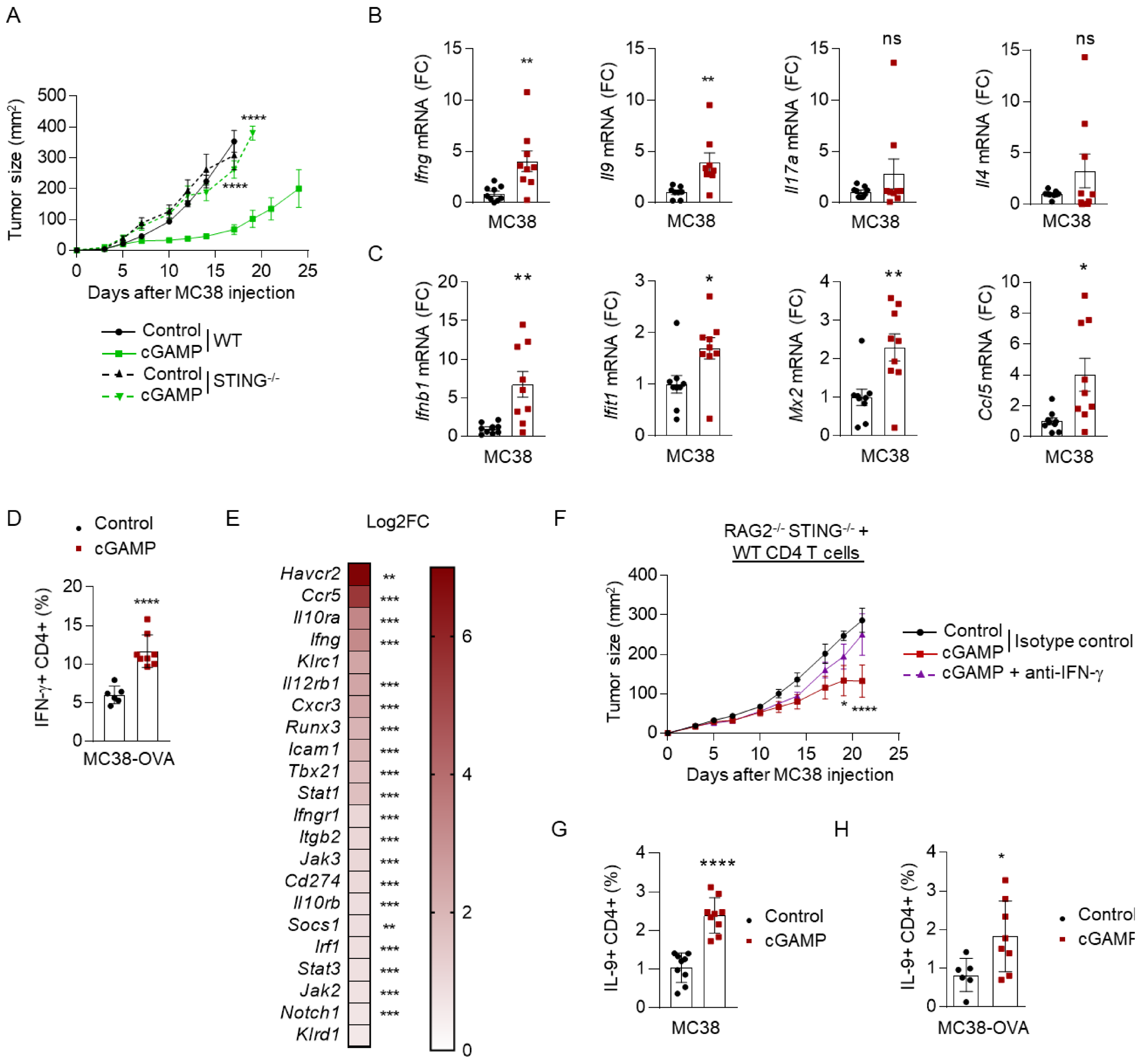

Benoit-Lizon I, *et al. J Immunother Cancer* 2022; 10:e003459. doi: 10.1136/jitc-2021-003459

Figure S2: The antitumor effect of cGAMP *in vivo* requires STING and modulates CD4 T cell effector functions

(A) Tumor size in MC38 tumor-bearing WT or STING$^{-/-}$ mice treated or not *i.t.* with cGAMP. Mean ± SEM of n=8-10 mice per group pooled from two independent experiments.

(**B**) *Ifng, Il9, Il17a and Il4* mRNA relative expression (FC) in MC38 tumor-infiltrating immune cells, after *i.t* cGAMP treatment.

(C) *Ifnb1*, *Ccl5*, *Ifit1* and *Mx2* mRNA relative expression (FC) in MC38 tumor-infiltrating CD4 T cells, after *i.t* cGAMP treatment.

Mean ± SD from n=8-9 mice per group pooled from three independent experiments.

P values (**p*<0.05, ***p*<0.01, *****p*<0.0001) determined by two-way ANOVA **(A)** or unpaired t tests **(B and C)**.

(**D, H**) IFN-γ (**D**) and IL-9 (**H**) production in TILs from MC38-OVA tumor-bearing WT mice after *i.t.* cGAMP treatment and stimulated *ex vivo* with class II-restricted OVA-peptide (323-339). Mean ± SD of technical duplicates from n=3-4 mice.

(E) Heatmap illustrating the relative expression of T_H1-related genes between CD4 T isolated cells from STING V154M/WT mice and their control littermates. Mean Log2FC from biological replicates (n=5 mice per group). Adjusted *p* values (***p*<0.01, ****p*<0.0001).

(**F**) MC38 tumor size in STING$^{-/-}$ RAG2$^{-/-}$ mice reconstituted with WT CD4 T cells and STING$^{-/-}$ CD8 T cells, and treated or not *i.t.* with cGAMP as well as *i.p.* with anti-IFN-γ. Mean ± SEM of n=8-9 mice per group pooled from two independent experiments.

(**G**) IL-9 production in TILs from MC38 tumor-bearing WT mice, after *i.t.* cGAMP treatment. Mean ± SD from three independent experiments where each dot represents one mouse (n=9 mice).

Benoit-Lizon I, *et al. J Immunother Cancer* 2022; 10:e003459. doi: 10.1136/jitc-2021-003459

Figure S3: STING ligands enhance the production of effector cytokines from in vitro-polarized T_H1 and T_H9 cells rather than affecting cell viability

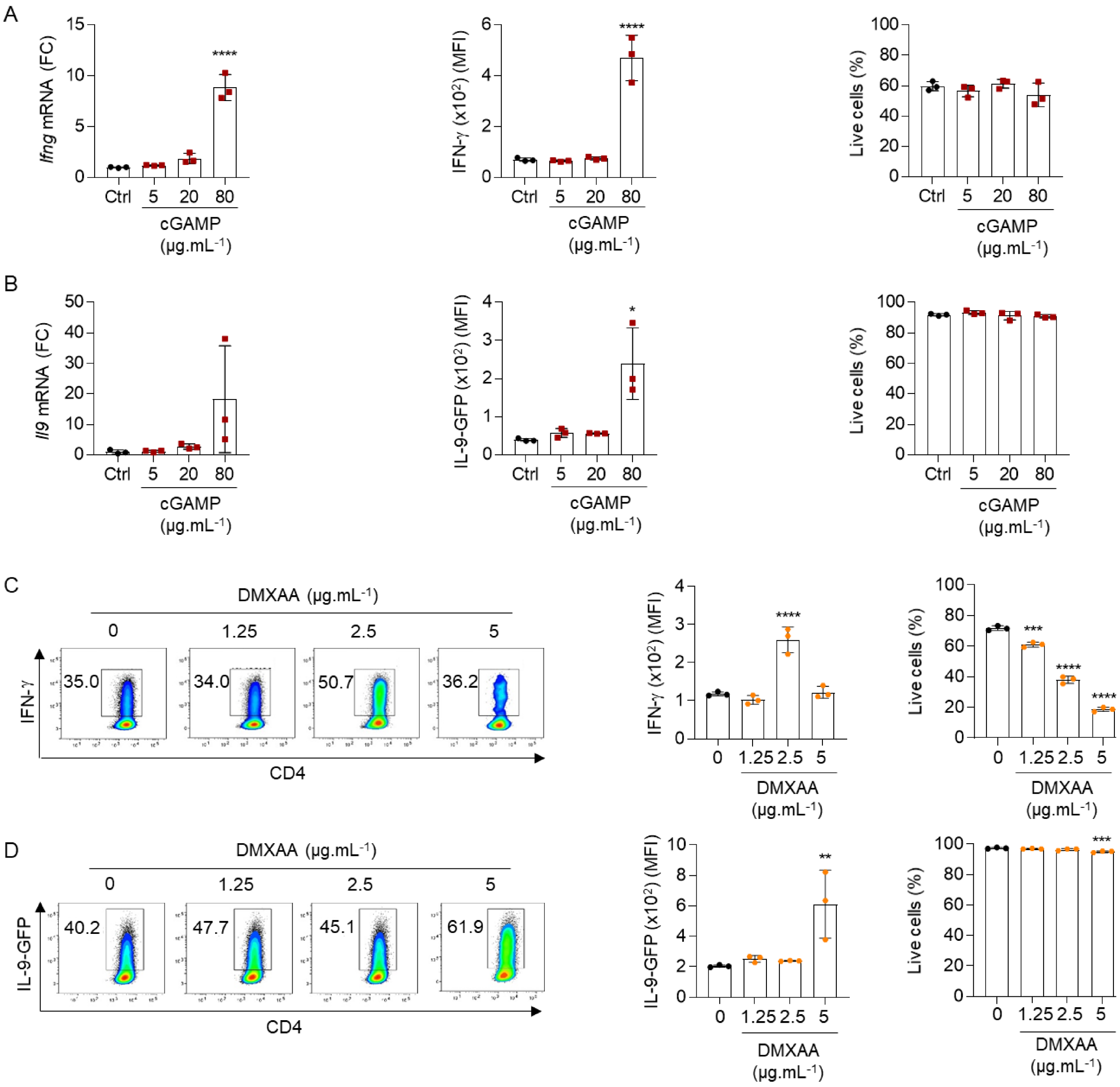

Benoit-Lizon I, *et al. J Immunother Cancer* 2022; 10:e003459. doi: 10.1136/jitc-2021-003459

Figure S3: STING ligands enhance the production of effector cytokines from *in vitro*-polarized T_H1 and T_H9 cells rather than affecting cell viability

(A, B) *Ifng* **(A)** or *Il9* **(B)** mRNA expression (FC, **left**), IFN-γ **(A)** or IL-9 **(B)** production (MFI, **middle**) and cell viability (**right**) from WT **(A)** or IL-9-GFP **(B)** naive CD4 T cells stimulated with cGAMP or Control (Ctrl) and polarized into T_H1 **(A)** or T_H9 **(B)** cells.

(C, D) IFN-γ and IL-9 production (representative plots (**left**); MFI (**middle**)) and cell viability (**right**) from WT **(C)** or IL-9-GFP **(D)** naive CD4 T cells stimulated with DMXAA or DMSO and polarized into T_H1 **(C)** or T_H9 **(D)** cells.

Mean ± SD of replicates from one experiment representative of three **(A, B)** or two **(C, D)** independent experiments. *P* values (*$p<0.05$, **$p<0.01$, ***$p<0.001$, ****$p<0.0001$) determined by one-way ANOVA.

Benoit-Lizon I, *et al. J Immunother Cancer* 2022; 10:e003459. doi: 10.1136/jitc-2021-003459

Figure S4: STING signaling is activated in CD4 T cells and maintained after 48h of polarization

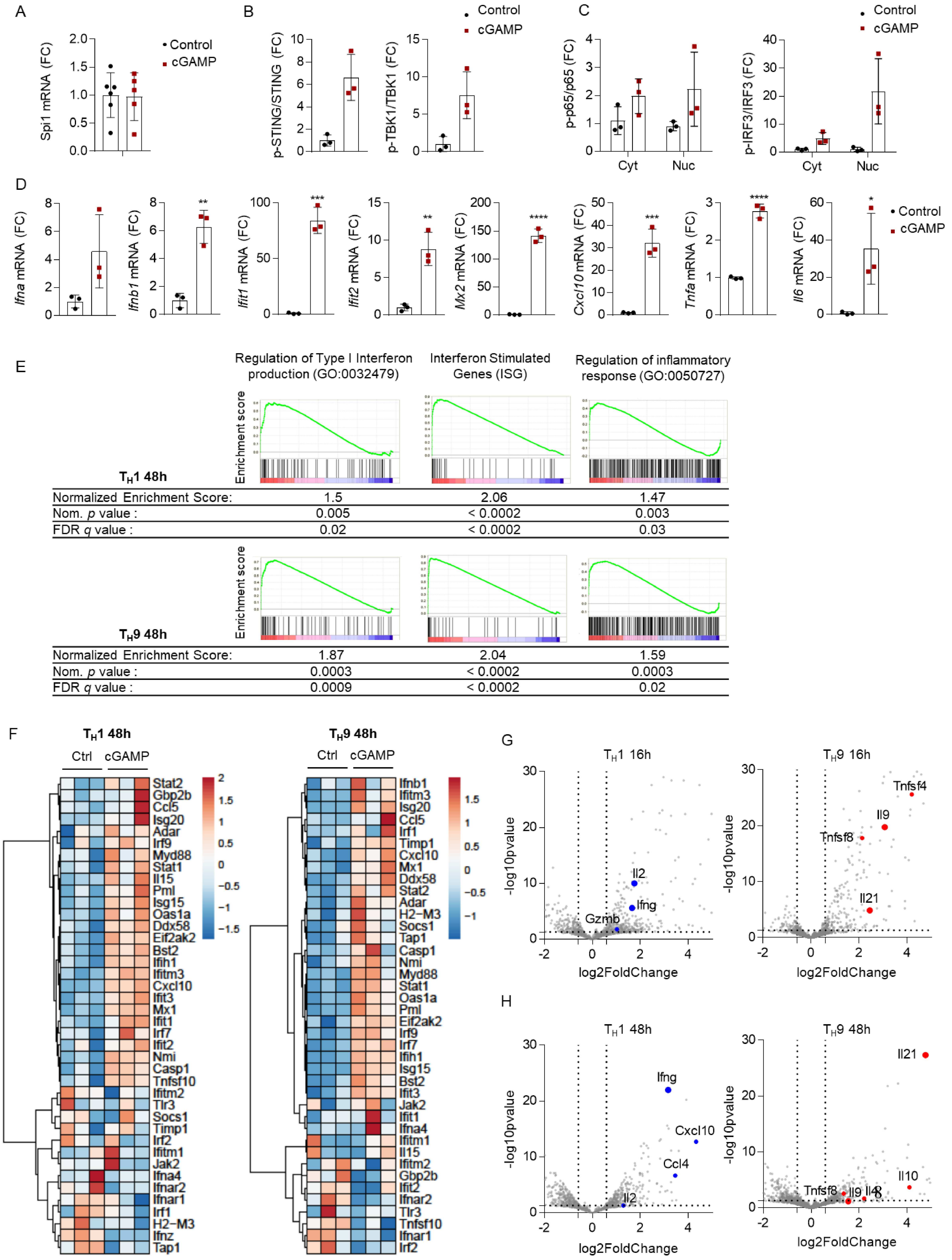

TH1 48h	Regulation of Type I Interferon production (GO:0032479)	Interferon Stimulated Genes (ISG)	Regulation of inflammatory response (GO:0050727)
Normalized Enrichment Score:	1.5	2.06	1.47
Nom. *p* value :	0.005	< 0.0002	0.003
FDR *q* value :	0.02	< 0.0002	0.03

TH9 48h	Regulation of Type I Interferon production (GO:0032479)	Interferon Stimulated Genes (ISG)	Regulation of inflammatory response (GO:0050727)
Normalized Enrichment Score:	1.87	2.04	1.59
Nom. *p* value :	0.0003	< 0.0002	0.0003
FDR *q* value :	0.0009	< 0.0002	0.02

Figure S4 I

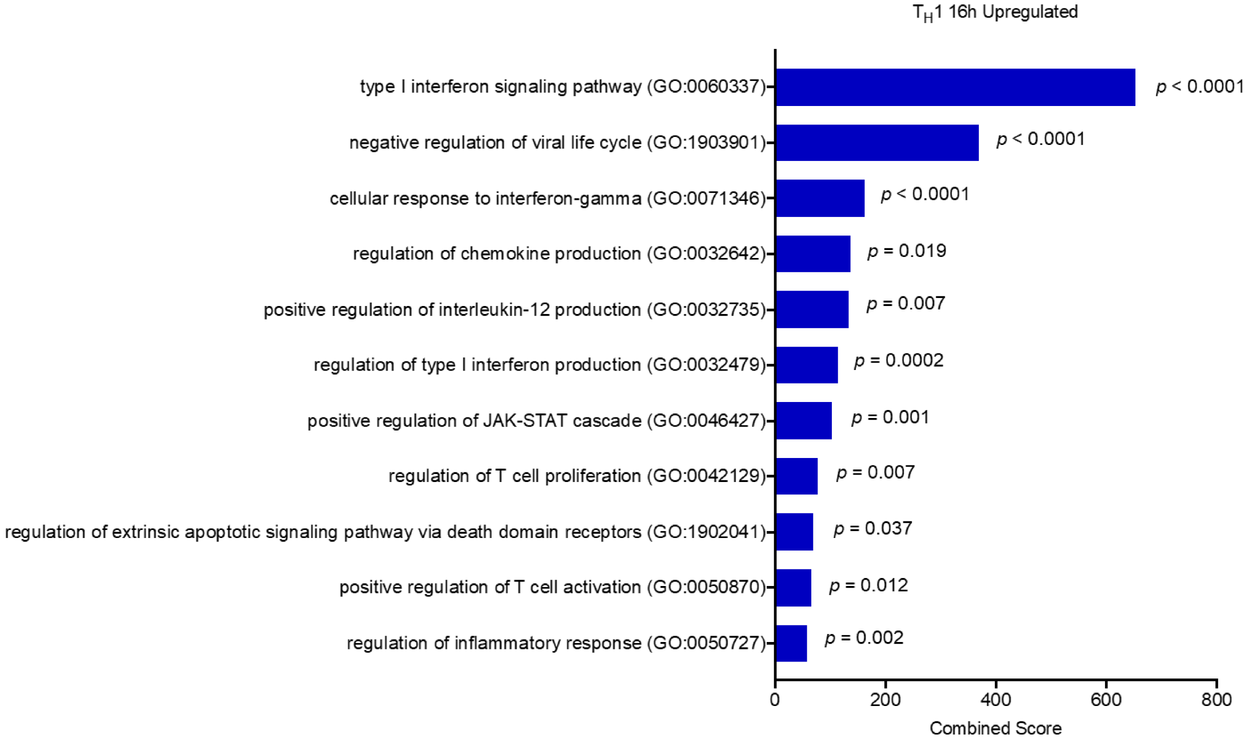
T_H1 16h Upregulated
type I interferon signaling pathway (GO:0060337)
negative regulation of viral life cycle (GO:1903901)
cellular response to interferon-gamma (GO:0071346)
regulation of chemokine production (GO:0032642)
positive regulation of interleukin-12 production (GO:0032735)
regulation of type I interferon production (GO:0032479)
positive regulation of JAK-STAT cascade (GO:0046427)
regulation of T cell proliferation (GO:0042129)
regulation of extrinsic apoptotic signaling pathway via death domain receptors (GO:1902041)
positive regulation of T cell activation (GO:0050870)
regulation of inflammatory response (GO:0050727)
p < 0.0001
p < 0.0001
p < 0.0001
p = 0.019
p = 0.007
p = 0.0002
p = 0.001
p = 0.007
p = 0.037
p = 0.012
p = 0.002
0
200
400
600
800
Combined Score

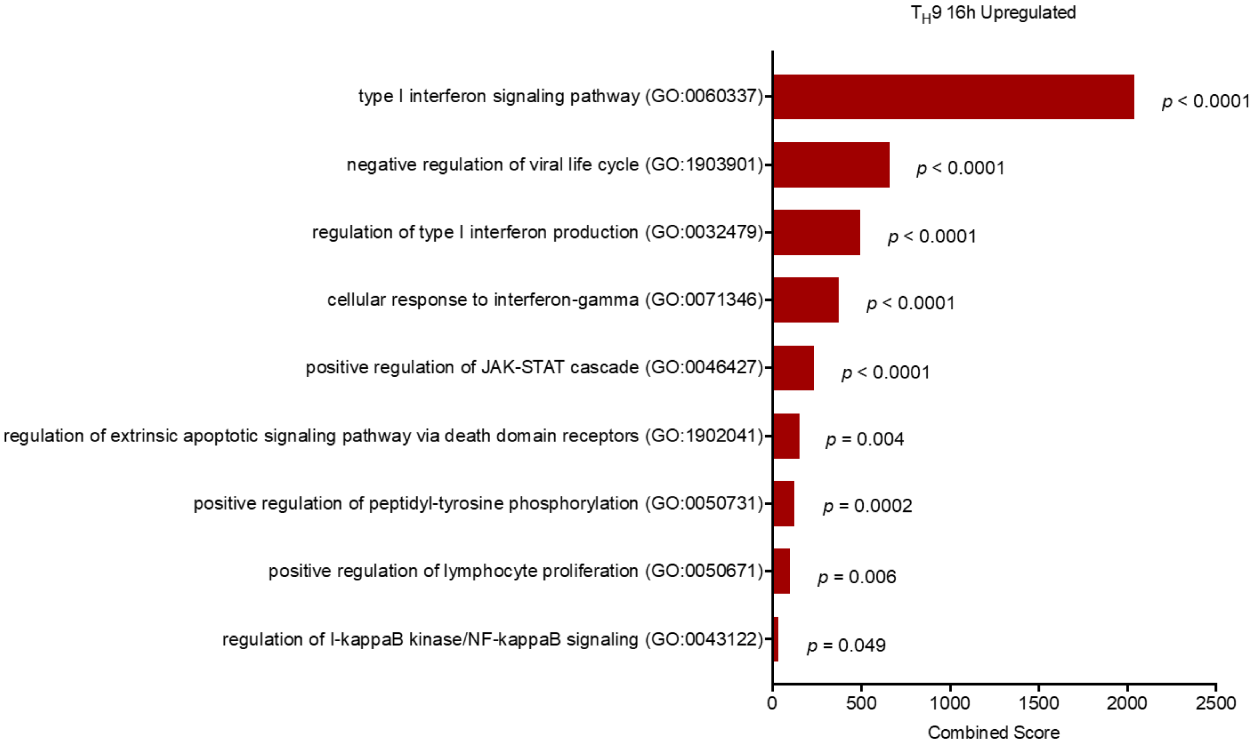
T_H9 16h Upregulated
type I interferon signaling pathway (GO:0060337)
negative regulation of viral life cycle (GO:1903901)
regulation of type I interferon production (GO:0032479)
cellular response to interferon-gamma (GO:0071346)
positive regulation of JAK-STAT cascade (GO:0046427)
regulation of extrinsic apoptotic signaling pathway via death domain receptors (GO:1902041)
positive regulation of peptidyl-tyrosine phosphorylation (GO:0050731)
positive regulation of lymphocyte proliferation (GO:0050671)
regulation of I-kappaB kinase/NF-kappaB signaling (GO:0043122)
p < 0.0001
p < 0.0001
p < 0.0001
p < 0.0001
p < 0.0001
p = 0.004
p = 0.0002
p = 0.006
p = 0.049
0
500
1000
1500
2000
2500
Combined Score

Figure S4 I

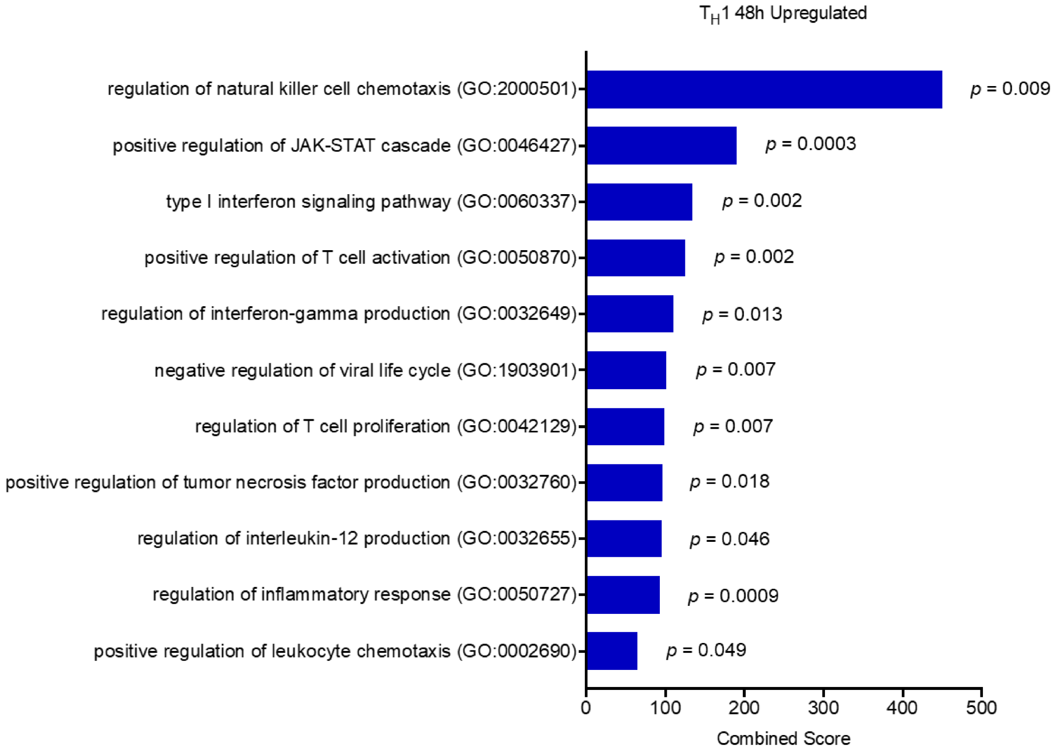
T_H1 48h Upregulated
regulation of natural killer cell chemotaxis (GO:2000501)
positive regulation of JAK-STAT cascade (GO:0046427)
type I interferon signaling pathway (GO:0060337)
positive regulation of T cell activation (GO:0050870)
regulation of interferon-gamma production (GO:0032649)
negative regulation of viral life cycle (GO:1903901)
regulation of T cell proliferation (GO:0042129)
positive regulation of tumor necrosis factor production (GO:0032760)
regulation of interleukin-12 production (GO:0032655)
regulation of inflammatory response (GO:0050727)
positive regulation of leukocyte chemotaxis (GO:0002690)
$p = 0.009$
$p = 0.0003$
$p = 0.002$
$p = 0.002$
$p = 0.013$
$p = 0.007$
$p = 0.007$
$p = 0.018$
$p = 0.046$
$p = 0.0009$
$p = 0.049$
0
100
200
300
400
500
Combined Score

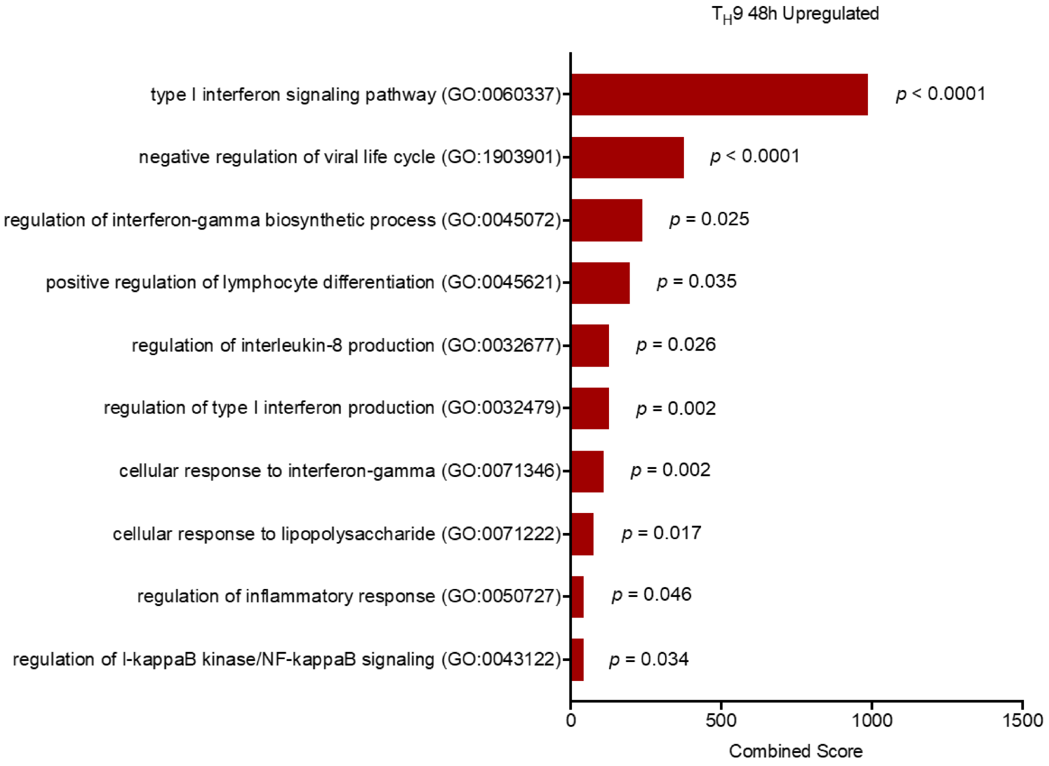
T_H9 48h Upregulated
type I interferon signaling pathway (GO:0060337)
negative regulation of viral life cycle (GO:1903901)
regulation of interferon-gamma biosynthetic process (GO:0045072)
positive regulation of lymphocyte differentiation (GO:0045621)
regulation of interleukin-8 production (GO:0032677)
regulation of type I interferon production (GO:0032479)
cellular response to interferon-gamma (GO:0071346)
cellular response to lipopolysaccharide (GO:0071222)
regulation of inflammatory response (GO:0050727)
regulation of I-kappaB kinase/NF-kappaB signaling (GO:0043122)
$p < 0.0001$
$p < 0.0001$
$p = 0.025$
$p = 0.035$
$p = 0.026$
$p = 0.002$
$p = 0.002$
$p = 0.017$
$p = 0.046$
$p = 0.034$
0
500
1000
1500
Combined Score

Figure S4: STING signaling is activated in CD4 T cells and maintained after 48h of polarization

(A) *Spi1* mRNA expression (FC) from naive CD4 T cells stimulated with cGAMP or Control and polarized into T_H9 cells for 16h.

(B, C, D) WT naive CD4 T cells stimulated with cGAMP or Control for 4h **(B)** or 6h **(C, D)**.

(B, C) Fold change (FC) of total STING, p-STING, TBK1 and p-TBK1 protein levels **(B)** and Cytosolic (Cyt) and nuclear (Nuc) localization of IRF3, p65 NF-κB and their phosphorylated forms **(C)**. Mean of replicates pooled from three independent experiments.

(D) *Ifna*, *Ifnb1*, *Il6*, *Tnfa*, *Ifit1*, *Ifit2*, *Mx2* and *Cxcl10* mRNA expression (FC). Mean ± SD of replicates from one experiment representative of three independent experiments.

P values (**p*<0.05, ***p*<0.01, ****p*<0.001, *****p*<0.0001) determined by unpaired t tests (**D**).

(E, F, G, H, I) RNA sequencing analysis from WT naive CD4 T cells stimulated with cGAMP or Control and polarized into T_H1 or T_H9 cells for 16h **(G-I)** or 48h **(E-F-H-I)**. Biological replicates from three independent experiments.

(E) Gene set enrichment analysis (GSEA) comparing expression in cGAMP-stimulated cells to Control cells. Enrichment plot and score, Nominal (Nom.) *p* value and False Discovery Rate (FDR) *q* value shown for the three gene sets. **(F)** Heatmaps illustrating the hierarchical clustering of expression levels (rld values) of Interferon Stimulated Genes (ISG). **(G-H)** Volcano plots depicting fold change (log2, x-axis) and statistical significance (−log10 adjusted *p* value, y-axis) in cGAMP-stimulated cells compared to Control cells. T_H1 (blue) and T_H9 (red) -related genes are highlighted. **(I)** Selection of biological pathways found significantly enriched in cGAMP-stimulated T_H1 and T_H9 cells compared to Control cells, using Enrichr web tool and GO

Biological Process 2018 database. Pathways ranked according to combined score. Adjusted *p* value indicated for each pathway.

Figure S5: STING-driven enhancement of T_H1 and T_H9 cell differentiation respectively involves IRF3 and mTOR signaling

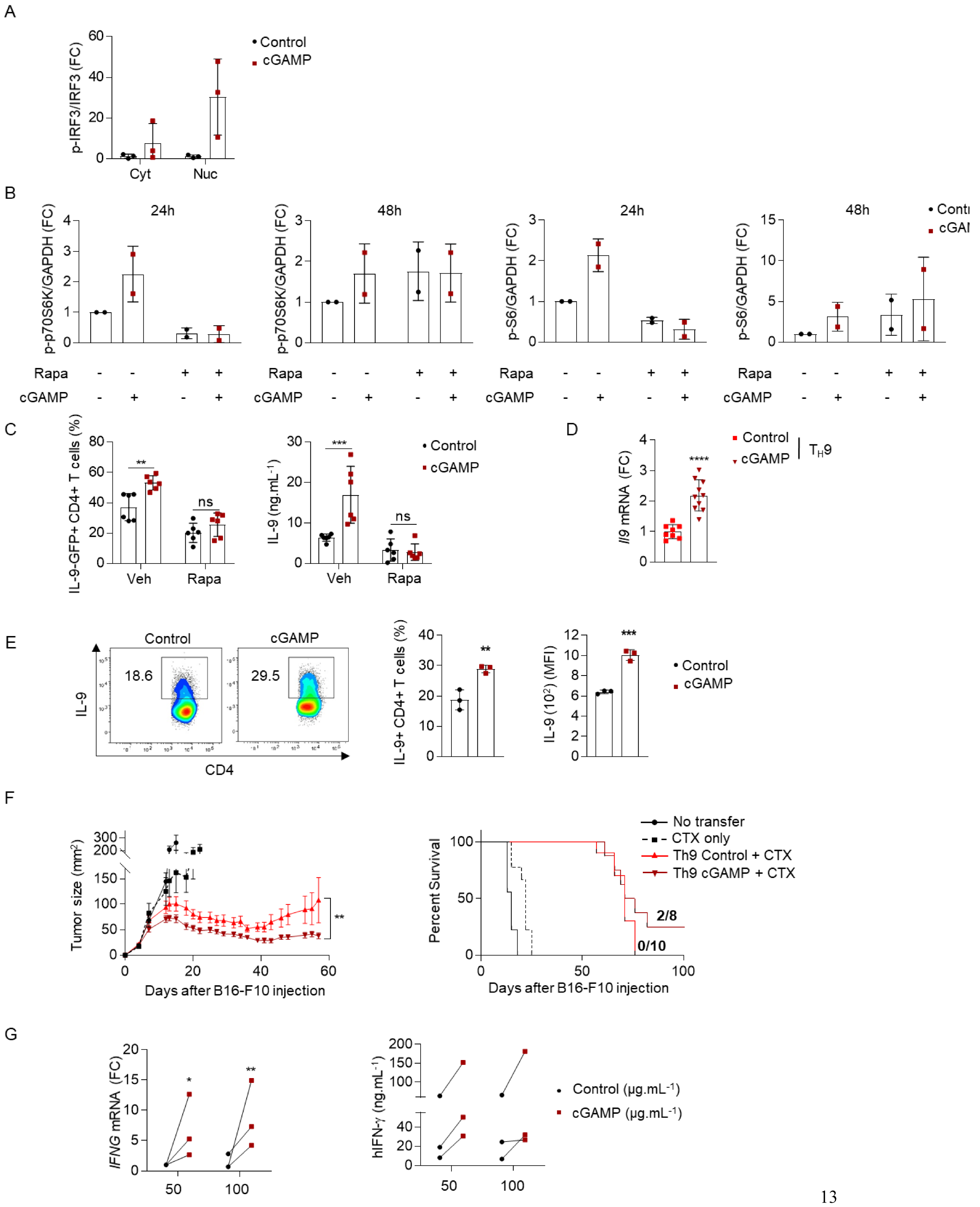

Benoit-Lizon I, et al. J Immunother Cancer 2022; 10:e003459. doi: 10.1136/jitc-2021-003459

Figure S5: STING-driven enhancement of T_H1 and T_H9 cell differentiation respectively involves IRF3 and mTOR signaling

(A) Fold change (FC) of cytosolic (Cyt) and nuclear (Nuc) localization of IRF3 and its phosphorylated form in WT naive CD4 T cells stimulated with cGAMP or Control and polarized into T_H1 for 16h. Mean ± SD pooled from three independent experiments.

(B, C) WT **(A)** or IL-9-GFP **(B)** naive CD4 T cells stimulated with cGAMP or Control in the presence of Rapamycin (Rapa, 10 nM, +) or vehicle (Veh, -) and then polarized into T_H9 cells for 24h and 48h **(A)** or 3 days **(B)**.

(B) Fold Change (FC) of phosphorylated p70S6K (P-p70S6K, **left**) and S6 (P-S6, **right**) levels. Mean ± SD pooled from two independent experiments.

(C) IL-9 production (frequency of IL-9-GFP+ CD4+ T cells, **left)** and IL-9 secretion (**right**). Mean ± SD of replicates pooled from two independent experiments.

(D) *Il9* mRNA expression (FC) from OT-II naive CD4 T cells stimulated with cGAMP or Control and polarized into T_H9 cells. Mean ± SD of 8-10 biological replicates pooled from two independent experiments.

(**E**) IL-9 production (representative plots (**left**); frequency (**middle**) and Mean Fluorescence Intensity (MFI, **right**)) from Trp-1 T_H9 cells differentiated in the presence of Trp-1 peptide-loaded $STING^{-/-}$ APC and stimulated with cGAMP (T_H9 cGAMP) or not (T_H9 Control). Mean ± SD of replicates from one representative experiment.

(**F**) Tumor size (**left**) and survival (**right**) in B16-F10 tumor-bearing mice treated or not with cyclophosphamide (CTX) before *i.v* injection of Trp-1 T_H9 cells differentiated in the presence of

Trp-1 peptide-loaded STING$^{-/-}$ APC and stimulated with cGAMP (T_H9 cGAMP) or not (T_H9 Control). Mean ± SEM of n=8-10 mice per group pooled from two independent experiments.

(G) *IFNG* mRNA expression (FC) **(left)** and hIFN-γ-secretion **(right)** from human naive CD4 T cells stimulated with cGAMP or Control and polarized into T_H1 cells. Mean of replicates from 3 independent experiments with each dot representing one donor.

P values (**p*<0.05, ***p*<0.01, ****p*<0.001, *****p*<0.0001) determined by two-way ANOVA **(C-H)** or unpaired t tests **(D)**

Supplemental Table 1: Primers and TaqMan assays used for mouse and human gene expression quantification

**Primers for mouse gene expression, SybR Green technology**		
**Target**	**Forward primer (5'-3')**	**Reverse primer (5'-3')**
Actb	atggaggggaatacagccc	ttctttgcagctccttcgtt
Ifng	gagctcattgaatgcttggc	gcgtcattgaatcacacctg
Il9	aacagtccctccctgtagca	aaggatgatccaccgtcaaa
Il17a	tgagcttcccagatcacaga	tccagaaggccctcagacta
Tbx21	atcctgtaatggcttgtggg	tcaaccagcaccagacagag
Irf1	aggcatccttgttgatgtcc	aattccaaccaaatcccagg
Gata3	aggatgtccctgctctcctt	gcctgcggactctaccataa
Irf4	caagcacagagtcacctgg	tgcaagctctttgacacaca
Irf8	aatgatgagaagaccatgttccgt	ccagcttgcatttttgttcttcct
Batf	gccccctagcagtcaagaag	tcagatgagtcctgtttgccag
Rorc	ggtgataaccccgtagtgga	ctgcaaagaagacccacacc
Foxp3	ctcgtctgaaggcagagtca	tggcagagaggtattgaggg
Spi1	tgcagctctgtgaagtggtt	agcgatggagaaagccatag
Ifna	gaatttcccctgacccagga	acttctgctctgaccacctc
Ifnb1	tccagcactgggtggaatga	ggtacctttgcaccctccagt
Ifit1	tcaaggcaggtttctgagga	attctctcccatggttgctgt
Ifit2	gctctggaaaaggacccgaa	gcttcagtgccaagaggact
Mx2	cctattcaccaggctccgaa	cagcataacctttgcgaaattct
Ccl5	ccacttcttctctgggttgg	gtgcccacgtcaaggagtat
Cxcl10	ctcatcctgctgggtctgag	cctatggccctcattctcac
Tnfa	agggtctgggccatagaact	ccaccacgctcttctgtctac
Il6	accagaggaaattttcaataggc	tgatgcattgcagaaaaca
**Primers for human gene expression, SybR Green technology**		
ACTB	gttgtcgacgacgagcg	gcacagagcctcgcctt
IL9	catggctgttcacaggaaaa	ccatgcaaacaagataccca
IFNG	gtattgctttgcgttggaca	gagtgtggagagaccatcaagga
**TaqMan™ Gene Expression Assays**		
**Target**	**TaqMan Assay ID**	**Probe/Quencher**
Actb	Mm02619580_g1	FAM/MGB
Ifng	Mm01168134_m1	FAM/MGB
Il9	Mm00434305_m1	FAM/MGB
Il4	Mm00445259_m1	FAM/MGB
Il17a	Mm00439618_m1	FAM/MGB

Benoit-Lizon I, *et al. J Immunother Cancer* 2022; 10:e003459. doi: 10.1136/jitc-2021-003459

Supplemental Table 2: Genes most differentially expressed in cGAMP-stimulated T_H1 and T_H9 cells: For each time point and T cell subset, gene names, Log2 of fold change (Log2FC), adjusted *p*-value and *s*-value are represented. Top 30 genes ranked according to *s*-value.

T_H1 16h				T_H1 48h			
**Gene**	**Log2FC**	**Adj. *p*-value**	***s*-value**	**Gene**	**Log2FC**	**Adj. *p*-value**	***s*-value**
**Irf7**	5.14	3.54E-57	8.61E-39	**Ifi211**	134.03	5.87E-21	5.06E-14
**Ifi214**	3.51	1.03E-57	2.53E-32	**Ifng**	549.21	1.01E-22	2.80E-13
**Cxcl10**	6.87	1.06E-39	8.79E-32	**Cxcl10**	42.15	1.83E-13	1.84E-10
**Oasl2**	5.40	1.47E-40	7.20E-30	**Il10**	42.89	2.55E-11	6.09E-10
**Trim30a**	2.62	4.23E-64	1.26E-27	**Ifi209**	237.25	2.40E-16	7.50E-10
**Trim30d**	3.56	6.68E-45	3.11E-26	**Ermn**	202.60	8.52E-16	9.14E-10
**Ifi208**	3.64	4.65E-44	5.99E-26	**Ccl3**	37.34	1.09E-10	1.43E-09
**Rnf213**	2.85	4.81E-54	1.25E-25	**Ifi204**	57.90	8.21E-12	6.56E-09
**Ifi206**	3.97	2.34E-39	6.77E-25	**Frmd4b**	157.52	6.95E-14	2.32E-08
**Usp18**	3.82	4.78E-35	2.65E-22	**Ttc39c**	129.88	1.23E-13	3.42E-08
**Oas3**	3.80	4.98E-34	2.29E-21	**Mx1**	50.56	7.37E-12	4.46E-08
**Rsad2**	6.58	8.31E-26	5.41E-21	**P2ry14**	40.01	3.50E-10	1.78E-07
**Ifit3**	4.46	2.37E-28	4.48E-20	**Il22**	25.30	4.16E-06	2.73E-07
**Ccl5**	5.67	8.99E-24	1.83E-19	**Ifi214**	73.27	2.03E-10	5.34E-07
**Ifit1**	3.59	2.58E-31	4.71E-19	**Tnfrsf8**	156.24	2.50E-12	1.01E-06
**Ddx60**	3.80	3.64E-29	1.20E-18	**Rnf213**	958.60	9.66E-13	1.88E-06
**Mx1**	5.63	5.23E-22	7.20E-18	**Plac8**	618.77	3.78E-13	2.54E-06
**Ifi27l2a**	3.29	3.80E-29	4.34E-17	**Ccl4**	30.68	2.09E-07	3.99E-06
**Herc6**	2.90	3.15E-32	9.63E-17	**Ly6c1**	244.41	9.21E-11	6.44E-06
**Ifi44**	4.96	3.66E-23	1.52E-16	**Serpinb9**	231.41	1.14E-12	9.69E-06
**Ifi209**	2.58	3.94E-35	3.13E-16	**Trim30d**	218.92	2.07E-11	1.35E-05
**Ifih1**	2.96	9.53E-30	5.34E-16	**Ifih1**	155.49	1.83E-09	1.57E-05
**Xaf1**	3.86	3.86E-23	2.16E-15	**Parp12**	23.61	3.27E-06	2.07E-05
**Rtp4**	4.83	1.34E-18	1.19E-14	**Usp18**	234.60	1.73E-10	3.11E-05
**Ifi211**	3.27	7.65E-23	4.69E-14	**Lgals3bp**	113.85	4.26E-08	4.66E-05
**Plagl1**	2.91	8.17E-24	2.04E-13	**Oasl2**	43.13	5.10E-06	1.09E-04
**Slfn8**	2.47	2.37E-28	4.35E-13	**Ifi44**	39.10	6.13E-06	1.30E-04
**Oasl1**	5.98	1.52E-13	7.17E-13	**Ifi208**	28.97	7.58E-06	1.74E-04
**Isg15**	5.51	5.13E-15	9.93E-13	**Isg15**	53.30	1.73E-06	2.19E-04
**Slfn5**	2.80	2.67E-23	1.73E-12	**Slfn8**	87.56	1.05E-07	2.43E-04

Benoit-Lizon I, *et al. J Immunother Cancer* 2022; 10:e003459. doi: 10.1136/jitc-2021-003459

T_H9 16h				T_H9 48h			
**Gene**	**Log2FC**	**Adj. *p*-value**	***s*-value**	**Gene**	**Log2FC**	**Adj. *p*-value**	***s*-value**
**Gbp6**	4.86	4.92E-75	2.28E-51	**Ly6a**	4.82	5.53E-28	8.08E-20
**Irf7**	6.21	6.79E-64	1.15E-49	**Trim30d**	4.25	8.07E-29	8.14E-20
**Trim30a**	3.69	1.32E-80	2.68E-46	**Il21**	4.76	4.79E-28	8.47E-20
**Trim30d**	4.60	3.45E-56	1.76E-38	**Xaf1**	5.12	8.39E-20	6.25E-16
**Oas3**	4.65	2.28E-51	3.18E-35	**Oas3**	3.61	5.71E-23	6.53E-15
**Ifi27l2a**	3.62	4.10E-59	2.07E-34	**Trim30a**	3.18	1.56E-22	1.53E-13
**Ifi214**	4.10	3.70E-49	5.85E-32	**Ifih1**	3.07	6.29E-15	1.16E-09
**Ifih1**	4.42	5.48E-47	1.30E-30	**Irf7**	4.03	4.79E-12	2.39E-09
**Rnf213**	3.00	1.61E-59	1.06E-29	**Gbp6**	3.99	1.07E-09	1.60E-08
**Ccl5**	8.39	1.92E-34	6.81E-29	**Ifi44**	5.58	3.88E-08	5.32E-08
**Ifi209**	3.60	6.84E-47	7.74E-28	**Isg15**	4.65	4.85E-08	8.51E-08
**Ly6a**	4.58	6.86E-30	6.03E-22	**Tnfrsf8**	2.99	8.62E-11	1.61E-07
**Slfn5**	3.18	1.47E-39	1.43E-21	**Lgals3bp**	3.04	3.54E-09	5.19E-07
**Ifi206**	4.40	2.11E-30	3.34E-21	**Plac8**	4.91	5.84E-06	1.09E-06
**Ifi208**	3.85	2.71E-32	5.60E-21	**Inhba**	2.56	4.01E-10	1.88E-06
**Ifit3**	4.28	1.50E-29	7.27E-20	**Tlr7**	3.60	8.20E-08	3.15E-06
**Gbp9**	3.73	7.72E-28	1.76E-18	**Lilr4b**	2.75	1.16E-08	4.39E-06
**Tnfsf4**	4.19	2.49E-26	4.45E-18	**Usp18**	2.70	6.65E-09	5.55E-06
**Lgals3bp**	3.30	2.58E-30	7.67E-18	**Olr1**	4.82	9.20E-06	6.60E-06
**Oasl2**	5.89	1.45E-24	1.10E-17	**Oas1a**	4.95	3.22E-05	9.64E-06
**Parp14**	2.40	1.64E-41	2.86E-17	**Frmd4b**	3.17	2.59E-07	1.25E-05
**Ifi44**	5.21	1.77E-20	6.29E-17	**Rnf213**	2.26	1.16E-09	1.62E-05
**Rsad2**	5.19	2.97E-22	9.60E-17	**Irgm1**	2.24	6.82E-08	3.69E-05
**Xaf1**	4.68	9.83E-25	1.64E-16	**Il10**	4.09	2.07E-04	6.34E-05
**Ifi213**	4.11	1.46E-22	2.75E-16	**Ifi209**	3.06	3.28E-05	9.48E-05
**Usp18**	3.61	5.68E-26	3.91E-16	**Ifi211**	5.61	4.33E-04	1.26E-04
**Irgm1**	3.03	1.74E-29	5.15E-16	**Gbp2**	2.16	3.86E-07	1.65E-04
**Rtp4**	5.18	2.52E-20	6.66E-16	**Parp14**	1.97	5.25E-08	2.52E-04
**Iigp1**	4.14	1.30E-23	8.99E-16	**Ifi27l2a**	1.95	9.33E-08	3.47E-04
**Slfn8**	2.99	2.34E-28	1.38E-15	**Etv6**	1.76	1.16E-09	3.99E-04
